# Shift and homogenization of gut microbiome during invasion in marine fishes

**DOI:** 10.1186/s42523-022-00181-0

**Published:** 2022-06-04

**Authors:** Arthur Escalas, Jean-Christophe Auguet, Amandine Avouac, Jonathan Belmaker, Thanos Dailianis, Moshe Kiflawi, Renanel Pickholtz, Grigorios Skouradakis, Sébastien Villéger

**Affiliations:** 1 MARBEC, Univ Montpellier, CNRS, Ifremer, IRD, Montpellier, France; 2 The Steinhardt Museum of Natural History, Tel Aviv University, Tel Aviv-Yafo, Israel; 3 George S. Wise Faculty of Life Sciences, School of Zoology, Tel Aviv University, Tel Aviv-Yafo, Israel; 4 Institute of Marine Biology, Biotechnology and Aquaculture, Hellenic Centre for Marine Research, 71003 Heraklion, Greece; 5 The Department of Life Sciences, Ben Gurion University, 84102 Beer Sheva, Israel; 6 The Inter-University Institute for Marine Sciences, 88103 Eilat, Israel

**Keywords:** Gut bacteria, *Siganus*, Mediterranean Sea, Non-native species, Herbivorous fish, Homogenization

## Abstract

Biological invasion is one of the main components of global changes in aquatic ecosystems. Unraveling how establishment in novel environments affects key biological features of animals is a key step towards understanding invasion. Gut microbiome of herbivorous animals is important for host health but has been scarcely assessed in invasive species. Here, we characterized the gut microbiome of two invasive marine herbivorous fishes (*Siganus rivulatus* and *Siganus luridus*) in their native (Red Sea) and invaded (Mediterranean Sea) ranges. The taxonomic and phylogenetic diversity of the microbiome increased as the fishes move away from the native range and its structure became increasingly different from the native microbiome. These shifts resulted in homogenization of the microbiome in the invaded range, within and between the two species. The shift in microbial diversity was associated with changes in its functions related with the metabolism of short-chain fatty acids. Altogether, our results suggest that the environmental conditions encountered by Siganidae during their expansion in Mediterranean ecosystems strongly modifies the composition of their gut microbiome along with its putative functions. Further studies should pursue to identify the precise determinants of these modifications (e.g. changes in host diet or behavior, genetic differentiation) and whether they participate in the ecological success of these species.

**Supplementary Information:**

The online version contains supplementary material available at 10.1186/s42523-022-00181-0.

## Introduction

Biological invasions are among the biggest threats to ecosystems, with invasive species ranking first as driver of plant and animal extinctions [12]. Temperature tolerance, high fecundity, broad physiological and nutritional niches are the most often cited drivers of invaders ecological success [17, 30, 37, 83]. Recently, the recognition of the fundamental role played by the microbiome in the biology and fitness of the host [52, 53] has increased its consideration as a source of adaptive potential to new environmental conditions that could determine invasion success [36, 63]. For instance, it was shown that modifications of plant microbiome can enhance host fitness in the invaded range [7, 11]. However, there is only few studies on the microbiome of marine invaders [23, 26, 41], and there are even fewer that compared their microbiome in both the native and invaded range [32, 67], which is the first step towards better understanding of how living in novel areas impact gut microbiome and eventually the invasion success.

The Mediterranean Sea is considered the region most heavily impacted by exotic species [24], most of which originate from the Red Sea and entered the Eastern Mediterranean Sea through the Suez Canal [29]. Among these Lessepsian invaders, two herbivorous fishes from the Siganidae family (i.e. rabbitfishes), *Siganus rivulatus* and *Siganus luridus* are particularly successful and are thus listed among the 100 “worst invasive” alien species threatening biodiversity in Europe [74]. Since their establishment, in 1924 and 1955, respectively [10, 73], their range has expanded westward to reach the Tunisian coasts and northward to the North of Aegean Sea [42]. While representing < 10% of fish assemblages biomass until the 1980s, they currently account for 30–40% in many places, exceeding 50% in some localities [65]. The herbivory pressure associated with this success has reduced the complexity of the invaded ecosystems, notably the biomass of canopy-forming algae (− 65%), benthic algae and invertebrates (− 60%) and overall species richness (− 40%, [69, 77, 78]. This constitute a regime shift from seagrass beds and habitat-forming seaweeds to “turf” dominated ecosystems [65, 82]. In addition, *S. rivulatus* and *S. luridus* have been shown to serve as bioinvasion vectors for dozens of benthic non-indigenous species in the Mediterranean Sea thanks to live passage through their digestive tracts [33].

**Fig. 1:**
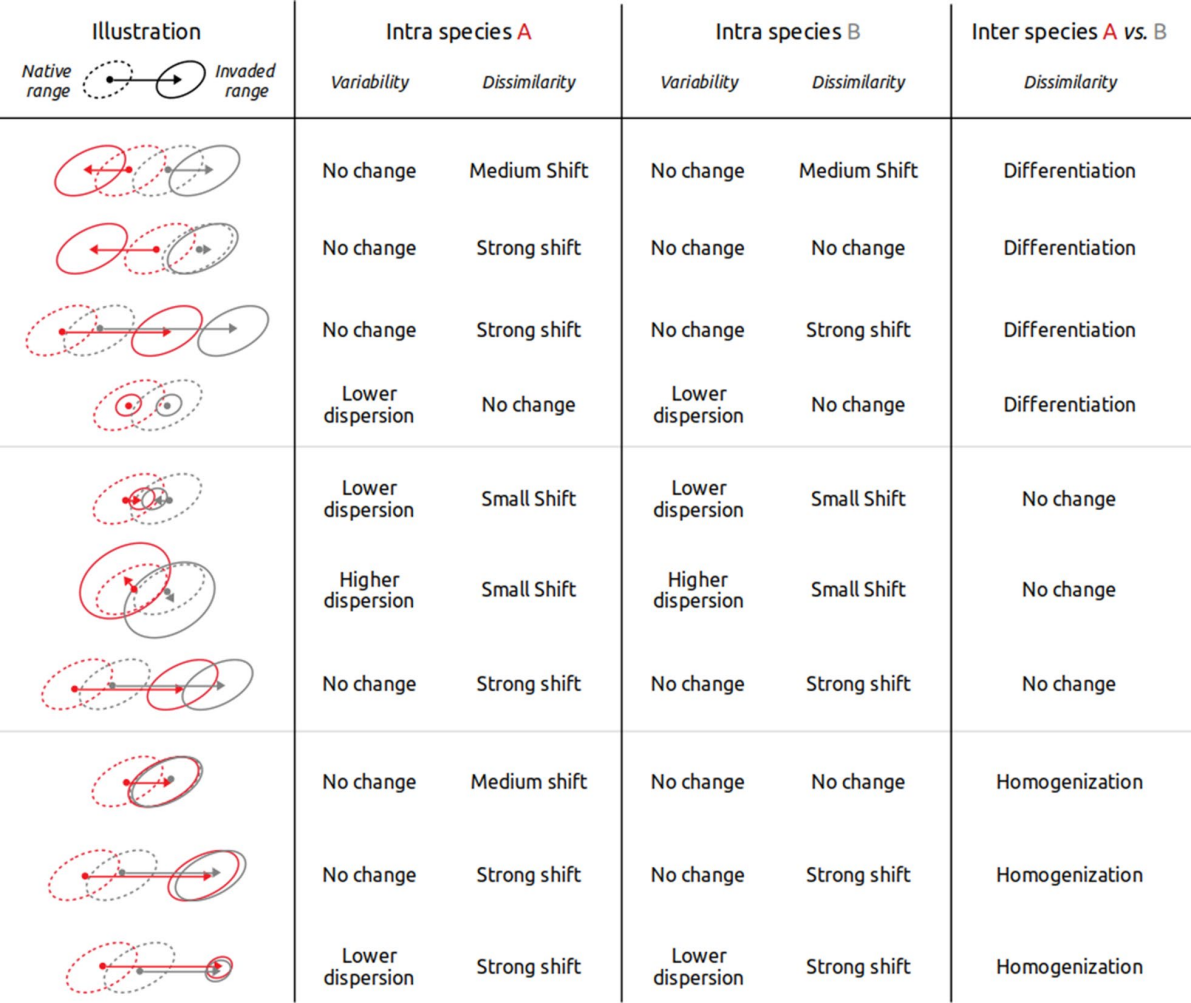
Possible trajectories of microbiome modifications during invasion. This figure presents different possible trajectories of the microbiome of two invasive species (A in red and B in grey) in their native (dashed ellipses) and non-native (continuous ellipses) ranges. Each ellipse represents the dissimilarity in microbiome composition between individuals from a given species and population, as often represented in an ordination. The microbiome of each species can become more or less variable in the non-native range, as depicted by the size of the ellipses, while the non-native microbiome can be more or less dissimilar to the native one, as depicted by the arrows and the degree of overlap between native and non-native ellipses. Ultimately, these intra-specific microbiome modifications could result in differentiation or homogenization of the microbiome between the species. These examples highlight the possibility of a similar inter-species outcome arising from different combinations of intra-specific trajectories. This framework can be applied to study inter-specific modifications of other ecological traits than the microbiome (i.e. diet breadth, isotopic niche, morphological or behavioral traits) and in other contexts than invasions (e.g. response to disturbance)

The importance of microbiome in host health appears particularly high for herbivorous hosts, which rely on microorganisms to extract nutrients from hard-to-digest, nutrient poor and sometimes chemically defended food sources [34, 45, 61]. Several groups of microorganisms are known for their contribution to fermentative processes allowing vertebrate herbivores (not only fishes) to extract nutrients from their nutrient-poor vegetal food sources. In fishes, these include notably members of the Firmicutes (e.g. *Epulopiscium*, Erysipelotrichaceae, Ruminococcaceae, [55, 57]) and members of the *Rikenella* genus, that are known to degrade complex algal polysaccharides, such as cellulose, into short-chain fatty acids (SCFA) available for the host through microbial fermentation [20, 54]. In herbivorous fishes, the production of SCFAs is particularly important for the host nutrition and results from high fermentation rates within the hindgut [21, 22]. During the colonization-establishment-invasion process, invaders face novel environmental conditions, food sources and inter-specific interactions (e.g. feeding on novel food sources such has seagrass beds and macroalgae). All these changes could lead the invaders to behavioral and/or dietary modifications, which may influence how they interact with micro-organisms and ultimately impact their gut microbiome in different ways (Fig. 1, [14, 50]. For instance, the diversity of gut microbiome in each individual could change during the invasion process due to host genetic bottleneck or reduced population size that limit horizontal transfer of microorganisms, or due to modification of diet breadth in the invaded range. Then, dissimilarity of the microbiome between individuals could decrease (i.e. intraspecific homogenization), or increase (i.e. differentiation), along the invasion gradient following, for instance, diversification or specialization of the diet in the non-native range. In addition, these intraspecific changes could be associated with changes in the dissimilarity between species, leading to interspecific homogenization or differentiation of the microbiome in the invaded range.

In this study, we aim to determine the types of microbiome modifications within and between two *Siganus *species during their invasion of the Mediterranean Sea. Microbiome of each species can indeed increase in diversity during invasion with varying levels of dissimilarity compared to native populations and such changes could lead to differentiation or homogenization between the two species (Fig. 1). For that, we assessed the differences in the gut microbiome of both *S. rivulatus* and *S. luridus* from their native range (Northern Red Sea) and in two sites in the Eastern Mediterranean Sea where they have successfully established (Levantine Sea and Northern Crete). By characterizing different facets of their microbiome (alpha and beta taxonomic, phylogenetic and functional diversity), we highlight strong modifications associated with range expansion, leading to a homogenization of the microbiome both within and between species in the non-native range.

## Material and methods

### Samples collection

Samples were collected in three regions. Both *S. rivulatus* and *S. luridus* were sampled in their native range in the Northern Red Sea (Eilat, Israel), as well as their non-native range in the Levantine Sea and the Northern Crete. Two sampling campaigns were led per region, one in late Spring (June 2018 and 2019 in Israel and Crete, respectively) and one in early Autumn (October 2018 and 2019 in Israel and Crete, respectively). Weather before and during the sampling were typical of the studied areas (i.e. no flood or heatwaves). In each region, two to four distinct sites were selected to cover the various types of habitat structure and macrophytes communities observed locally (Additional file 1: Table S1 and S2).

Fish were collected in shallow habitats (2–10 m) by scuba divers using handnets and gillnets. Only adult individuals with a standard length > 100 mm were collected, hence ontogenetic variability of the microbiome between early life and adult stages was not addressed. Fish were immediately euthanized, stored on ice and processed less than two hours after capture. Fish were sized (mm), weighted (g) and dissected using tools cleaned with 70° ethanol. The last third of the gut (i.e. hindgut) was squeezed out on a piece of parafilm to collect the transient microbiome developing of food [56]. Contamination between samples was minimized by cleaning of dissection tools between each individual. The gut content was homogenized before storage at − 80 °C in a 3 mL cryotube until DNA extraction.

Microbial communities from the surrounding environment (i.e. water, sediment, and potential food sources: macrophytes, seagrasses and turf) were collected at the same time and in the same locations. Water samples were collected at 1 m below the surface using 500 mL plastic bottles. Planktonic microbes were collected on 0.2 µm GTTP filters (Whattman). Surface sediment samples were collected in 3 mL cryotubes. Macroalgae, seagrasses and turf growing on rocks and hard substrates were collected in individual plastic bags. These samples were rinsed with deionized water to remove interstitial seawater and rubbed using buccal swabs (Cliniscience). Filters, cryotubes and swabs were immediately stored on ice after sample collection and stored at -80 °C until DNA extraction.

A total of 356 microbiomes were analyzed, including 157 fishes (125 *S. rivulatus* and 32 *S. luridus*), 56 macroalgae, 29 seagrass, 31 sediment, 43 turf and 40 water samples (Additional file 1: Table S2).

### DNA extraction

DNA extractions were performed in the molecular biology platforms of the MARBEC laboratory (Montpellier, France, www.umr-marbec.fr/) and at the Genseq platform (genseq.umontpellier.fr), using the Qiagen MagAttract PowerSoil DNA KF Kit, selected for its compliance with the Earth Microbiome Project [51]. Extractions were performed in 96 well plates in which 3 wells were left empty to serve as negative controls and 3 wells were loaded using standard mock communities (ZymoBIOMICS Microbial Community DNA Standards II, Zymo Research). These standards of known composition were used to evaluate the quality of our sample processing pipeline. Extraction wells were loaded using half of a GTTP filter for water samples, half of a swab for turf samples, and ~ 0.25 g of gut content and sediment samples. DNA extraction protocol included a bead beating step and a chemical lysis. DNA recovery was based on magnetic beads and automated with a Kingfisher Flex robot. DNA was eluted in 100µL of elution buffer before quantification of DNA quantity and quality using a Nanodrop 8000 spectrometer. Extracted DNA was stored at 4 °C until PCR amplification, which was done the next day.

### PCR amplification

PCR amplification was done using universal bacterial primers selected for their compliance with the Earth Microbiome Project [60]: 515F-Y (5′-GTGYCAGCMGCCGCGGTAA) and 926R (5′-CCGYCAATTYMTTTRAGTTT). The targeted sequence was 411 bp and corresponded to the V3-V4 regions of the prokaryotic bacterial 16S rRNA gene. PCR amplification was carried out in 96 well plates in triplicate for each DNA extract and was done in a 25 µL reaction volume. The PCR mix consisted of 9.75 µL of water, 0.75 µL of DMSO, 0.5 µL of each primer, 12.5 µL of Phusion ready-to-use Taq mix (Phusion High-Fidelity PCR Master Mix with GC Buffer) and 1 µL of DNA. After an initial denaturation of 30 s at 98 °C, the PCR cycle consisted of 30 cycles of 10 s denaturation at 98 °C, 1 min annealing at 58 °C and 1 min 30 s of extension at 72 °C. Final extension was held for 10 min at 72 °C before keeping the reaction at 4 °C. The success of PCR amplification was checked using GelRed™ on 2% agarose gel in TAE buffer and using a 100 bp DNA ladder. The wells left empty during DNA extraction served as negative controls for contamination of the PCR reactions. PCR triplicates were pooled and stored at − 20 °C before sequencing. An amplicon library was constructed by the Genotoul platform (get.genotoul) and sequencing was carried out using an Illumina MiSeq (2 × 250 bp) sequencer in two separate sequencing runs.

### Amplicon sequencing and sequences processing

Reads from NGS were processed with the R software environment (v 4.1.2) using the package *dada2* v 1.19.2 [15]. Briefly, the quality of the reads for each sample was inspected using graphic representations of their quality scores and reads shorter than 240 bp and contained bases with a quality < 20 were discarded. Amplicon sequence variants (ASVs) were inferred using the dada algorithm (Divisive Amplicon Denoising Algorithm) after pooling dereplicated reads from all samples. Then, forward and reverse reads were merged and chimeric sequences were removed. The taxonomic classification of ASVs was performed with the naive Bayesian RDP classifier implemented in *dada2* and using the SILVA reference database nr_V132 (10.5281/zenodo.1172783). The quality of taxonomic assignment was assessed by bootstrap and ASVs with an assignment bootstrap value < 90 were discarded. As many ASVs were not affiliated at the genus level, ASVs sequences were blasted against the NCBI 16S rRNA bacterial database, and the best hit was used to correct ASV taxonomy [19]. We used percentage of similarity with the best hit sequence at 97% and 95% for species and genus level assignment, respectively [8]. ASV with matching score lower than these thresholds were left with unassigned genus and/or species.

Three data cleaning steps were performed. First, the mock communities and blank samples were used to identify contamination from the reagents (e.g. extraction kit, polymerase), and these ASVs were removed from the data set (e.g. *Ralstonia*, *Rhizobium*). Second, ASVs not assigned to the bacterial domain, unclassified at the Family level or above, or assigned to chloroplasts and mitochondria were filtered out. Third, ASVs present in 3 samples or less were removed.

Then, two datasets were used for subsequent analyses. The first dataset contained the samples from all compartments, it was rarefied at 2000 sequences per sample and was used to compare the diversity and composition of bacteria between ecosystems compartments. The second dataset contained only the gut microbiome of Siganus and was used for the analysis of the composition of the core gut microbiome along with predictions of its functional potential (see below). This later was not rarefied to keep as much information as possible.

### Identification of the core microbiome of *Siganus*

To compare the microbiome of *Siganus* between their native and non-native range, we focused our analyses on the most prevalent bacterial taxa. Hence, the core microbiome of each *Siganus* species was identified separately within each region using the core identification algorithm from [49]. This method accounts for both ASVs occurrence and abundance across communities and is based on the comparison of the observed abundance-occurrence distribution of ASVs with a random distribution under a stochastic Poisson model. For each species, we had a different number of individuals per region and to avoid biases related with sample size, we used a bootstrap approach in which we identified core ASVs for each species in each region using an equal number of samples corresponding to the minimal number of individuals sampled per region for each species (i.e. n = 32 for *S. rivulatus* and 9 for *S. luridus*). This procedure was repeated 1000 times using different combinations of individuals. Then, the core microbiome of *Siganus* used for analyses was defined as the ASVs identified as core in > 80% of the iterations in at least one region and one species. ASVs sequences of the fish core microbiome were aligned using *mafft* implemented in *Qiime2* before being inserted in the Greengene reference phylogenetic tree [39]. The tree was then ultrametricized using *pathd8* (http://www2.math.su.se/PATHd8).

### Inference of potential microbiome functions

The functions potentially performed by the gut microbiome of *Siganus* were identified using the functional inference approach from *Tax4Fun2* package v 1.1.5 [79] and a similarity percentage of 90% with the reference database. From all the KEGG Orthologies (KOs) availale from *Tax4Fun2* outputs we kept only the KOs that were related to the metabolism of short-chain fatty acid (SCFA). SCFA are the result from the fermentative processes occurring in the gut of herbivorous fishes and are considered as crucial for their nutrition [21, 22]. We considered SCFA composed of one to five C atoms: formate (1 C, n = 25 KOs), acetate (2C, 17 KOs), propionate (3C, 13 KOs), (iso)butyrate (4C, 17 KOs), (iso)valerate (5C, 14 KOs). Hereafter, abundance of KOs related to SCFA within each fish gut were treated like taxonomic diversity (i.e. KOs as distinct units).

### Data analysis

The diversity of microbial communities was assessed using the Hill numbers framework [18], which allows assessing alpha (*i.e*. within community) and beta (*i.e*. dissimilarity between pairs of communities) diversity indices for both taxonomic and phylogenetic facets of biodiversity using a single mathematical framework. In addition, Hill numbers indices can account for taxa/lineages/KOs presence or abundances, depending on the value of the parameter *q*. Here we estimated richness-like indices based on presence-absence data of taxa and lineages (*q* = 0), and entropy-like indices accounting for the relative abundance of taxa and lineages (*q* = 1). Similarly, we estimated dissimilarity in the composition of taxa (*i.e*. taxonomic dissimilarity), lineages (i.e. phylogenetic dissimilarity) and KOs using presence-absence data (*q* = 0) and dissimilarity in taxonomic and phylogenetic structure using relative abundance (*q* = 1).

We tested for difference between regions in the abundance of taxa and KOs using ANOVA on centered log-ratio transformed data (CLR, [31]. Differences in alpha diversity between ecosystem compartments (fish, sediment, water, macrophytes and turf), regions (North Red Sea, Levantine Sea and Northern Crete) and seasons (spring and autumn) were tested using ANOVA or Kruskall-Wallis tests. Differences in multivariate dispersion in the microbiome of between regions and seasons were tested using PERMDISP [2] and the *betadisper* function from the R package *vegan v 2.7* [59]. Differences in composition and structure of the microbiome between compartments, regions and seasons were tested using PERMANOVA [2] and the *adonis2* function from the R package *vegan* [59]. Pairwise differences between groups of samples were tested using the *pairwise.adonis2* function (https://github.com/pmartinezarbizu/pairwiseAdonis). Differences in the relative abundances of individual taxa and KOs between groups of samples were tested using Kruskall-Wallis tests and p-values were corrected using the FDR method as implemented in the *p.adjust* function from the *stats v 4.1.2* package. All these analyses were performed for three levels of bacterial taxonomic resolution (Phylum, Family and ASV) and at the KO levels for functional analyses of SCFA metabolism.

## Results

### Structure, diversity and determinants of marine ecosystem microbiomes

**Fig. 2:**
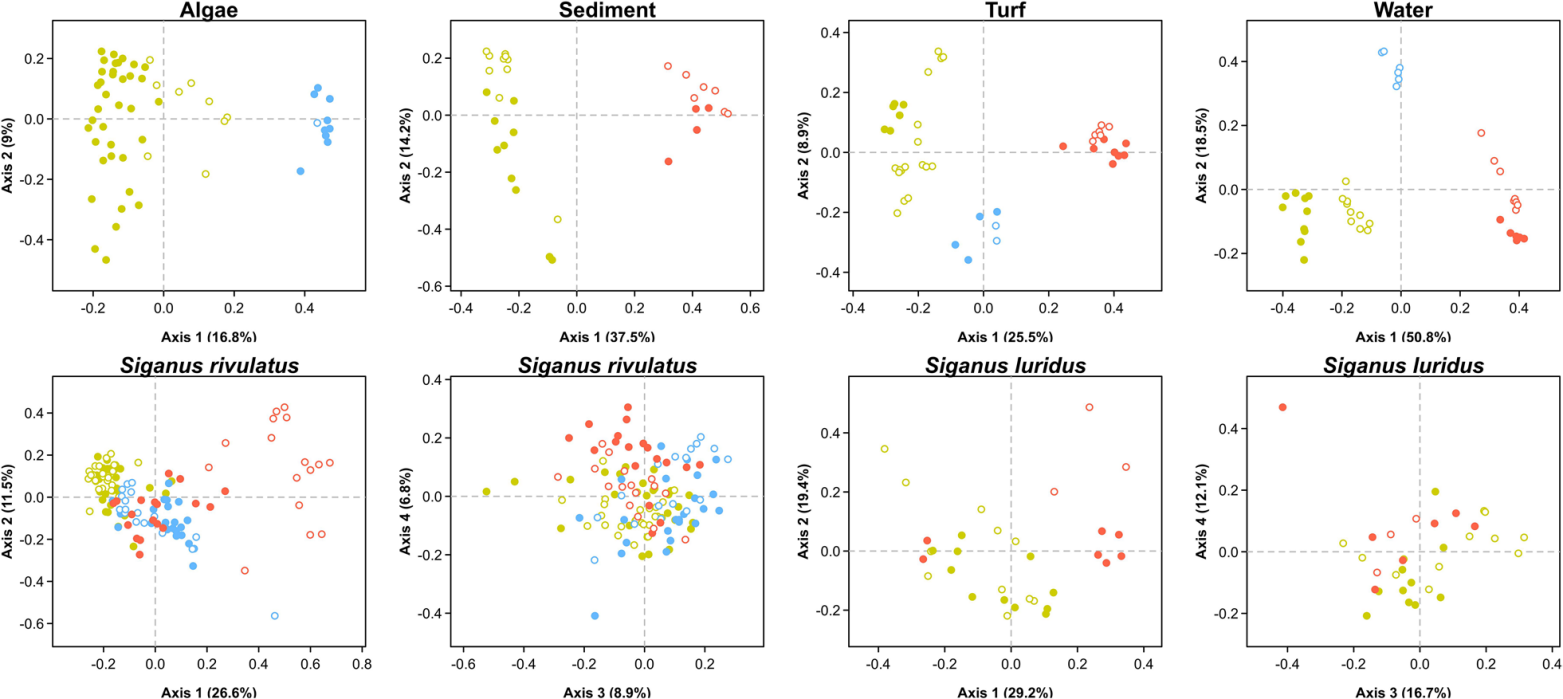
Regional and seasonal differences in the structure of environmental and fish gut microbiomes. Principal Coordinates Analyses (PCoA) were performed on abundance-weighted taxonomic dissimilarity computed on microbial ASVs (partitioning of taxonomic entropy expressed as Hill numbers). For each ecosystem compartment, red, blue and green dots correspond to samples from Red Sea, Levantine Sea and Northern Crete, respectively, with filled and empty dots correspond to spring and autumn, respectively. For gut microbiomes of both Siganidae species (bottom row), the first two pairs of PCoA axes are represented (PC1-PC2 and PC3-PC4)

Bacterial communities from all ecosystem compartments (water, sediment, algae, seagrasses, turf and fish) were dominated by two phyla (Additional file 1: Table S3), Proteobacteria (32 to 57% of the sequences) and Bacteroidetes (23 to 32%). Among Proteobacteria, the Alphaproteobacteria and Gammaproteobacteria classes were the most abundant in environmental compartments (13–41% and 11–25%, respectively) but contributed to only 1% of the fish microbiome, where they were replaced by Deltaproteobacteria (31% in the fish and 1–11% in other compartments, Additional file 1: Table S4). As a result, the structure of bacterial communities differed significantly between compartments (PERMANOVA, p-value < 0.05) for all the taxonomic ranks considered (Phylum, Family, ASVs). For all ecosystem compartments, microbiome structure differed mostly between regions (PERMANOVA, p-values < 0.05, Table 1 and Additional file 1: S5, Fig. 2 and Additional file 1: S1), as the F-values of this factor were 2 to 5 times higher than those of the season factor.

**Table 1:** Determinants of the structure of environmental and fish gut microbiomes

Microbiome	Factor	Number of significant tests	Average R^2^	Average F-value	F_region_/F_season_
*Taxonomic diversity*					
Algae	Region	4	0.09	6.0	2
	Season	4	0.05	3.2	
	Region:Season	1	0.01	1.0	
Sediment	Region	6	0.38	22.7	5
	Season	4	0.07	4.6	
	Region:Season	2	0.05	2.7	
Turf	Region	6	0.28	8.7	2
	Season	5	0.06	3.7	
	Region:Season	6	0.11	3.8	
Water	Region	6	0.48	35.0	2
	Season	6	0.09	15.3	
	Region:Season	5	0.07	9.2	
*S. luridus*	Region	6	0.33	18.2	8
	Season	1	0.04	2.2	
	Region:Season	6	0.13	9.5	
*S. rivulatus*	Region	6	0.32	30.3	6
	Season	4	0.02	5.0	
	Region:Season	6	0.17	31.1	
*Phylogenetic diversity*					
*S. luridus*	Region	6	0.38	21.6	12
	Season	0	0.03	1.8	
	Region:Season	6	0.15	11.8	
*S. rivulatus*	Region	6	0.36	36.6	9
	Season	3	0.01	4.3	
	Region:Season	6	0.20	40.7	

PERMANOVA tests were performed only for the microbiome types for which were sampled for at least two regions and two seasons (Region:Season corresponds to the effect of the interaction). The reported values correspond to averages of tests results performed across three taxonomic ranks (Phylum, Family and ASV) for taxonomic and phylogenetic dissimilarity, estimated with presence-absence or relative abundances data (i.e. *q* = 0 and 1, respectively). A more comprehensive table summarizing all the performed PERMANOVA (all combinations of dissimilarity indices and taxonomic ranks) is provided in additional file (Additional file 1: Table S13)

The differences in microbiome structure were associated with significant differences in alpha diversity between ecosystem compartments (Additional file 1: Figure S2, Kruskall-Wallis test, p-values < 0.001, Additional file 1: Table S6 and S7). Water microbial communities had the lowest ASVs richness (405 ± 135 ASVs), followed by fish gut microbiome (509 ± 153) and seagrass (518 ± 150), while algae (647 ± 138), sediment (615 ± 130) and turf (724 ± 152) communities had a higher ASVs richness. Alpha diversity (i.e. richness and entropy) of water and turf microbiomes differed significantly only between regions (ANOVA, p-values < 0.05, F-values = 15.8 and 10.4, respectively, Additional file 1: Table S8). Sediment microbiome diversity was influenced by both region and season (ANOVA, p-value < 0.05, F = 26.3 and 10.3, respectively).

### Structure and diversity of *Siganus* gut microbiomes differ between native and invaded ranges

The core microbiome of the two *Siganus species* comprised 704 ASVs that belonged to nine bacterial phyla, 11 classes, 19 families and 29 genera (Additional file 1: Figure S3). Three phyla dominated the core microbiome, Firmicutes, that were the most diverse and the second most abundant (239 ASVs and 22% of the relative abundance), along with Proteobacteria (175 ASVs, 41%) and Bacteroidetes (155 ASVs, 21%). The remaining phyla were Tenericutes (89 ASVs, 5%), Fusobacteria (9 ASVs, 6%), Verrucomicrobia (15 ASVs, 2%) and to a lesser extent Epsilonbacteraeota, Deferribacteres and Spirochaetes (< 11 ASVs, < 2%). The core microbiome represented a decreasing proportion of the total microbiome from the native to the invaded range in *S. rivulatus* but not in *S. luridus* (Additional file 1: Figure S6, Table S9 and S10). The two species of *Siganus* hosted different gut microbial communities overall (PERMANOVA, p-value < 0.001), including in the two regions where they co-occur (i.e. Red Sea and Northern Crete, Additional file 1: Table S11–S13). In addition, we did not observe any significant differences between fish sampled in different sites within each of the three regions (PERMANOVA, p-value > 0.05, Additional file 1: Table S14).

The structure of the gut microbiome of both *Siganus* species differed significantly between their native (Northern Red Sea) and non-native (Levantine Sea and Northern Crete) ranges (Table 1 and Fig. 2). More precisely, all PERMANOVAs based on taxonomic and phylogenetic abundance-weighted dissimilarity (*q* = 1) were significant (Table 1 and Additional file 1: Table S15). PERMANOVAs based on compositional dissimilarity indices (*q* = 0) were significant only for *S. rivulatus*, with the exception of a significant region effect at the ASV level for *S. luridus*. PERMANOVA computed at the phylum and family levels yielded similar conclusions.

The region factor explained a higher proportion of the variance in gut microbiome structure (R^2^) than the season factor and the F-values were much higher (6 to 9 times higher for *S. rivulatus* and 8 to 12 times higher for *S. luridus*, Table 1). A significant interaction between region and season factors was found for all abundance-weighted dissimilarity indices in *S. luridus* and for all indices in *S. rivulatus*. For this latter species, the microbiomes of fishes sampled in Red Sea in Autumn was distinct from other sets of fishes (at both the ASV and family level, Additional file 1: Figure S1). Pairwise comparisons revealed that gut microbiomes of *S. rivulatus* from Northern Crete and Red Sea were the most distinct and that gut microbiome from Northern Crete and the Levantine Sea were more distinct than those from the Red Sea and the Levantine Sea (Additional file 1: Table S16).

Similarly, the region factor had the strongest influence on taxonomic and phylogenetic richness and entropy (Additional file 1: Figure S2 and Table S17), with F-values 2.9 times higher on average than those of the season factor (Additional file 1: Table S18). For *S. rivulatus*, the lowest richness and entropy values were observed in the native range (North Red Sea), followed by the Levantine Sea and Northern Crete (ANOVA, p-values < 0.05, Additional file 1: Table S18). For *S. luridus* there was no significant difference in microbiome diversity between individuals from the Red Sea and from Northern Crete. In addition, we did not find significant difference in alpha diversity between fishes sampled in different sites within regions (Additional file 1: Table S19).

### Change in taxonomic composition led to microbiome homogenization in the non-native range

**Fig. 3:**
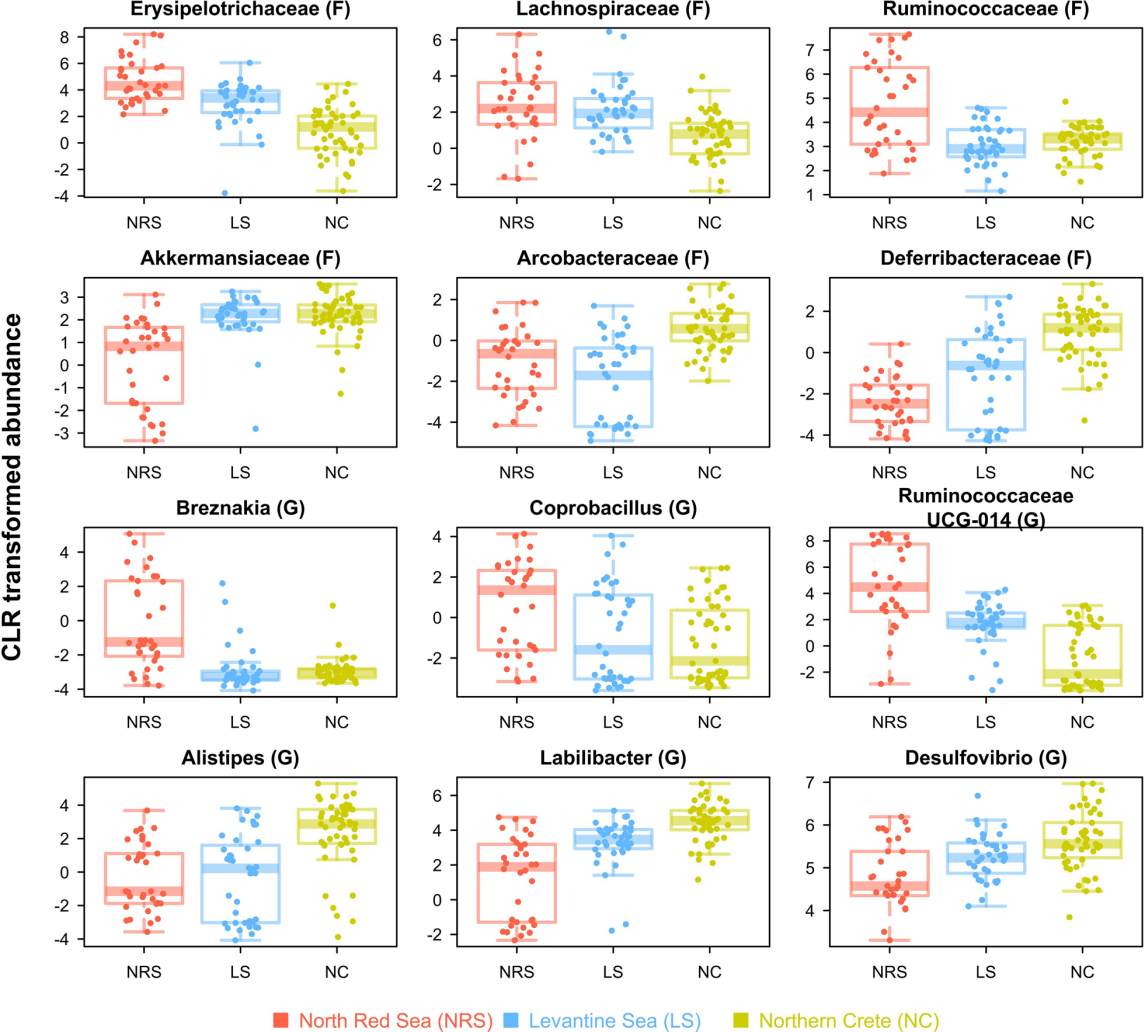
Bacterial taxa with the highest contrast in abundance in the gut of *S. rivulatus* across the 3 regions. Names of taxa are provided at top of each panel with letter in parentheses for taxonomic level (F: Family and G: Genus)

The abundance of many bacterial taxa changed between regions in both* Siganus* species, but significant differences were mostly observed for *S. rivulatus* (Fig. 3). For this species, 79% of the ASVs (i.e. 555/704) had significantly different abundances between regions (p-value < 0.05, Kruskal–Wallis test). The percentage was 89% at the phylum level (8/9), 79% at the family level (15/19) and 79% at genus level (23/29) (Additional file 1: Tables S20 and S21). The proportions were much lower for *S. luridus*, with 20% of the ASVs having a significantly different abundance between regions (142/704), 22% of the phylum (2/9), 37% of the families (7/19) and only three genera out of 29 (3%).

The most impacted phylum was the Firmicutes, which relative abundance decreased from 48% in Red Sea, to 29% and 12% in Levantine Sea and Northern Crete, respectively, for *S. rivulatus* and from 48 to 12% between native and invaded range for *S. luridus*. Three families in particular decreased in abundance in the non-native range in both species, with many genera having all their ASVs significantly differing in abundance between regions (Additional file 1: Table S20 and S21). First the Erysipelotrichaceae (Erysipelotrichia class), which 50 ASVs not assigned at the genus level decreased in abundance in *S. rivulatus*, along with the genera *Breznakia* (6 ASVs) that dropped from 2.5 to 0.01%, and *Coprobacillus* (3 ASVs) that dropped from 0.9 to 0.2%. Secondly, the Ruminococcaceae (Clostridia) were also strongly impacted, with 86% of unassigned ASVs (60/70) having different abundances between ranges, along with the 42 ASVs composing the dominant genus UCG-014 decreasing in abundance in the non-native range (29–0.6%), while other genera had only one ASV but were also differently abundant (*Faecalibacterium*, *Paludicola*). Thirdly, the Lachnospiraceae (Clostridia), with the prominent genera *Epulopiscium* (13 ASVs, 10% in Levantine Sea and < 0.5% in other regions), *Bacteroides* (3 ASVs, 2.6% to 0.4%) and *Tyzerella* (2 ASVs, 0.6% to 0.4%). The genera *Bacteroides, Tyzerella* and *UCG-014* were also impacted in *S. luridus*. Another taxa decreased in abundance in the invaded range, the Tenericutes phylum, shifted from 14% in Red Sea to 3% in Northern Crete.

These taxa were replaced by Proteobacteria, that increased from 17% in Red Sea to 31% in Levantine and reached 47% in Northern Crete for *S. rivulatus*, and shifted from 30 to 66% in *S. luridus*. Among these, the genus *Desulfovibrio* (Deltaproteobacteria, 52/79 significant ASVs) was the most prominent, shifting from 15% in Red Sea to 27 and 36% in Levantine Sea and Northern Crete, respectively. Similarly, Bacteroidetes increased from 15 to 25% between native and invaded range, respectively, but we observed contrasted results in this phylum. Indeed, while the Rikenellaceae family increased from 12% in Red Sea to reach a maximum of 17% in Levantine Sea, some of its genera were more abundant in the native (*Rikenella*, with 38/52 ASVs with significant differences) and others in the invaded range (*Alistipes* with 12/16 ASVs, *gut groups RC9* with 11/11 and *dgA-11* with 6/7). Among the Bacteroidetes, the genus *Labilibacter* increased from 3 to 14% between the native and invaded range (14/19 ASVs). Among the Verrucomicrobia, the genus *Akkermansia* shifted from 1.6% to > 5.8% in the invaded range, for both species (11 and 7 out of 15 ASVs with differential abundance in *S. rivulatus* and *S. luridus*, respectively).

**Fig. 4:**
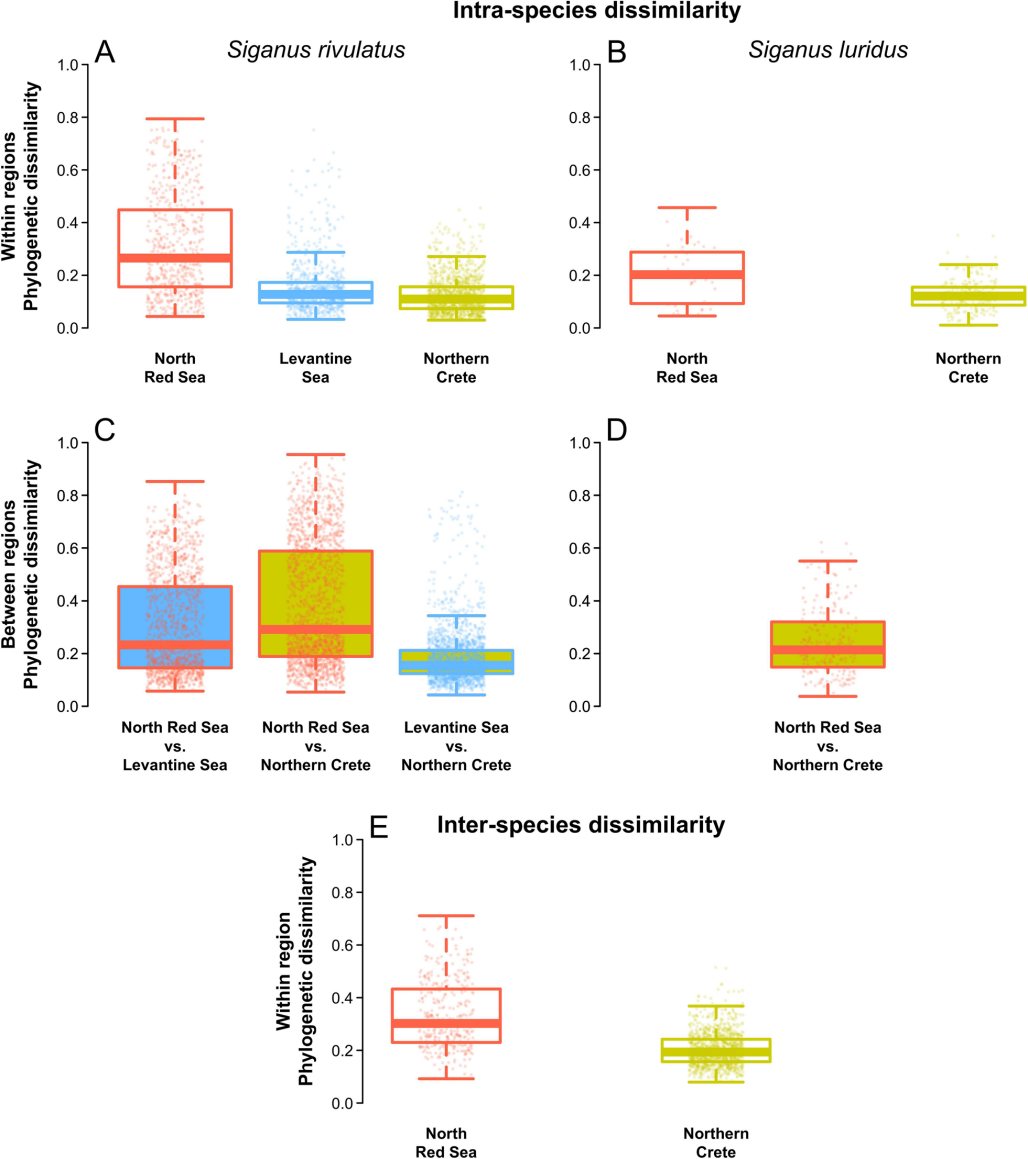
Phylogenetic dissimilarity of the gut microbiome of *Siganus *across their native (Red Sea) and invaded range (Mediterranean Sea: Levantine Sea and Northern Crete). The top row represents intraspecific dissimilarity of the gut microbiome among *S. rivulatus* (**A**) or *S. luridus* (**B**) individuals, within each of the regions where samples were collected. The middle row represents intraspecific dissimilarity between *S. rivulatus* (**C**) or *S. luridus* (**D**) individuals, between the regions where they were sampled. The bottom row (**E**) represents interspecific dissimilarity between *S. rivulatus* (**A**) and *S. luridus* (**B**) individuals, in the two regions where they were sampled simultaneously. Phylogenetic dissimilarity was computed on relative abundance of ASVs

All these changes in gut microbiome structure resulted in significant intraspecific homogenization within the invaded range compared to the native range for both species (Fig. 4A, B, p-value < 0.001, Kruskal–Wallis test, Additional file 1: Table S22 and Figure S8). Dissimilarity between *S. rivulatus* individuals decreased by 46 ± 14% on average (i.e. across the various indices and taxonomic ranks) from Red Sea to the Levantine Sea and by 61 ± 11% from Red Sea to Northern Crete. For *S. luridus*, the drop in dissimilarity was of 29 ± 9% between Red Sea and Northern Crete. On the contrary, none of the other compartments of the ecosystem showed homogenization between the Red and Mediterranean Sea (Additional file 1: Figure S9 and Table S22).

Furthermore, the microbiome of the two species became more similar in the non-native range (p-value < 0.001, Wilcoxon test, Additional file 1: Table S23), with a 32 ± 13% decrease in phylogenetic dissimilarity between the Red Sea and Northern Crete, on average (Fig. 4E). This significant loss was found for all taxonomic and phylogenetic dissimilarity indices (i.e., with or without considering taxa relative abundances, and for three levels of taxonomic resolution; Additional file 1: Figure S10 and Table S23).

### Shift in the taxonomic structure of the microbiome translates in change in its potential functional role

The functional structure of the gut microbiome significantly differed between the native and non-native ranges for both *Siganus* species (p-values < 0.001, PERMANOVA, Table 2). The proportions of KOs with significantly different abundance between regions varied among the 5 SCFA considered (Additional file 1: Table S24, Figure S11). For instance, in *S. rivulatus*, propionate had the highest proportion of differently abundant KOs (77%, 10/13) and the strongest inter-region differences (Kruskal–Wallis statistic = 20.3), while acetate showed the lowest proportion (35%, 6/17) and weakest effect (statistic = 9.5). Differences between SCFA were less marked in *S. luridus* (Additional file 1: Table S24, Figure S11). Pairwise comparisons in *S. rivulatus* revealed that the functional potential of the gut microbiome differed mostly between the population from native range (Red Sea) and the two populations established in the Mediterranean Sea (Levantine Sea and Northern Crete) (Additional file 1: Table S25).

**Table 2:** Determinants of the functional potential of *Siganus* gut microbiomes

SCFA	# of C atom	Factor	*Siganus rivulatus*				*Siganus luridus*			
			R^2^	*F* value	*F*_*region*_/*F*_*season*_	*p* value	R^2^	*F* value	*F*_*region*_/*F*_*season*_	*p* value
Formate	1	Region	0.42	44.0	2	0.001	0.36	17.0	43	0.001
		Season	0.14	19.6		0.001	0.01	0.4		0.913
		Region:Season	0.72	60.1		0.001	0.49	9.0		0.001
Acetate	2	Region	0.16	11.3	6	0.001	0.39	19.5	7	0.001
		Season	0.01	1.8		0.231	0.09	3.0		0.136
		Region:Season	0.25	7.7		0.001	0.45	7.7		0.002
Propionate	3	Region	0.47	54.2	6	0.001	0.33	14.8	16	0.001
		Season	0.07	8.6		0.008	0.03	0.9		0.382
		Region:Season	0.72	59.6		0.001	0.43	7.1		0.004
Butyrate	4	Region	0.39	38.2	14	0.001	0.39	19.3	11	0.002
		Season	0.02	2.6		0.186	0.06	1.8		0.241
		Region:Season	0.38	14.7		0.001	0.51	9.8		0.004
Valerate	5	Region	0.37	35.2	27	0.001	0.40	19.8	8	0.002
		Season	0.00	1.3		0.985	0.07	2.3		0.18
		Region:Season	0.58	33.1		0.001	0.50	9.4		0.001
All SCFA	1–5	Region	0.42	44.6	6	0.001	0.37	17.8	17	0.001
		Season	0.06	8.0		0.004	0.03	1.0		0.336
		Region:Season	0.61	37.8		0.001	0.46	8.0		0.001

The reported values correspond to averages of tests performed using dissimilarity estimated with relative abundances of KEGG Orthologies associated with the metabolism of short-chain fatty acids (SCFA) with different number of C atoms

The taxonomic turnover in the microbiome between native and invaded range also resulted in the homogenization of the functional potential of the gut bacterial communities (Additional file 1: Table S26 and figure S12). In *S. rivulatus*, the loss of functional dissimilarity from Red Sea to the Levantine Sea and to the Northern Crete was, on average, 49 ± 28% and a 65 ± 35%, respectively. Such significant differences were found for each of the five sets of KOs (p-value < 0.01, PERMDISP). In *S. luridus* the pattern was more nuanced, with a homogenization in the metabolism of acetate and propionate when using presence-absence data only, and a no difference for the other SCFAs (Additional file 1: Table S26). Finally, inter-specific functional dissimilarity dropped by 50 ± 13% on average between the Red Sea and Northern Crete (Additional file 1: Table S27) and such convergence was observed for each of the five studied SCFA.

## Discussion

### Microbiome shift during invasion

Most studies on the microbiome of marine invaders focused on the comparison with native species [1, 28, 40, 48] and only few have compared invaders microbiome in both their native and invaded ranges [16, 32, 41, 76], often with contrasting results.

Here, we highlight strong modifications in the composition and structure of the gut microbiome of *S. rivulatus* and *S. luridus* between their native and invaded ranges. Fishes from the Siganidae family tend to expand their range from tropical to temperate waters, both in the Mediterranean Sea and along the Western Australian coasts [38, 82] and the adaptability of their microbiome to new environments might be key to their ecological success. *Siganus rivulatus* and *S. luridus* from Red Sea host gut microbiomes similar to the ones described for other Siganidae such as *S. fuscescens* from Australia [41, 58], *S. guttatus* from Vietnamese coasts [44] and *S. oramin* from South-Eastern China [81]. The dominance of Firmicutes, Proteobacteria and Bacteroidetes phyla, and notably of the Ruminococcaceae, Desulfovibrionaceae, Rikenellaceae families, was associated with an herbivorous diet in several other fish families [27, 55, 75].

The proportion of bacterial taxa belonging to the core microbiome decreased from the native to the invaded range and we observed increased richness and entropy in the microbiomes from the invaded range. This means that the core microbiome fades as the fishes move away from their native range even if the whole microbiome is being enriched in rare taxa not observed in the native populations. In *S. luridus*, the shift in microbiome structure is mostly due to changes in relative abundance within the core microbiome while in *S. rivulatus* the shift in the core microbiome structure is accompanied with a decrease in the proportion of the total microbiome represented by the core and its replacement by other taxa that were not present in the native range. These change might be driven by the novel environmental conditions in the invaded area, including different food sources. Interestingly, a reverse pattern was reported in *S. fuscescens* from the Western Australian coasts [41], where the core microbiome represented a higher proportion of the total microbiome at the edges of the range.

Furthermore, the turnover of bacterial taxa between native and invaded ranges led to strong modifications of the gut microbiome structure in the two studied species, which resulted in an increasing differentiation from the native microbiome with distance from the native zone. This is the second evidence of such microbiome shift during the invasion process in Siganidae [41] and a similar pattern was reported in ascidian [16, 32, 76]. Finally, we showed that the microbiome became increasingly more homogeneous as fishes move away from the native zone, both between individuals from the same species but also between individuals from different species. This was reported in the ascidian *C. oblonga*, with colonies separated by 500 km in the native range hosting distinct microbiomes while colonies separated by 2000 km in the invaded range hosting similar microbiomes [32]. The underlying mechanisms that yielded the reported significant shift in the Siganidae gut microbiome during the invasion of the Mediterranean Sea remain to be unraveled to determine whether they participate in the colonization success of the hosts.

### Winners and losers among microbial taxa during invasion

The bacterial phyla that decreased the most in abundance in the non-native range were the Firmicutes, with notably the Ruminococcaceae (UCG-014 genus), Erysipelotrichaceae (*Breznakia*) and Lachnospiraceae (*Bacteroides*, *Tyzerella*) families, and the Tennericutes. These taxa are well known members of herbivorous fish microbiome [27, 55, 68, 75] because of their ability to anaerobically ferment plant polysaccharides to short chain fatty acids (SCFAs: acetate, formate, butyrate, propionate, valerate) thus transforming complex and indigestible polysaccharides into energy sources that can be absorbed by the host [25, 66, 72, 80].

We observed a significant enrichment of the non-native microbiome in Deltaproteobacteria from the *Desulfovibrio* genus, Bacteroidetes from the *Allistipes* (Rikenellaceae) and *Labilibacter* genera, along with Verrucomicrobia from the *Akkermansia* genus. These taxa were also found to be associated with the nutrition and metabolism of SCFA in different hosts [13, 71]. Deltaproteobacteria from the Desulfovibrionaceae family and Rikenellaceae were found in higher abundances in the microbiomes of *S. fuscescens* sampled at the edge of their range (higher latitude), compared with individuals from the native range [41]. For the three species of *Siganus* (i.e. *S. fuscescens, S. rivulatus* and *S. luridus*), the increasing dominance of these taxa is associated with higher latitudes, more temperate waters and a higher availability of macroalgae and seagrasses compared with the tropical coral reef from which they originate [41]. Interestingly, the SCFAs content in the hindgut of *S. fuscescens* was quantitatively and qualitatively similar at the two edges of its range, revealing that the microbiome shift had no influence on the production of these compounds from the ingested food [41]. The remaining question here is whether these changes in microbiome structure reflect the adaptation of Mediterranean Siganidae populations to their novel environment and, if so, what are the underlying mechanisms.

### Drivers of microbiome shift in Mediterranean Siganidae

Several factors could explain the shift in microbiome observed here. A first explanation could be related to differences in host genetic background. For instance, it was shown that the microbiome diversity was lower in the invaded range for the ascidian *Clavelina oblonga* [32], but this likely resulted from the reduced host genetic diversity in the invaded range (i.e. a founder or bottleneck effect). There is no evidence of such genetic bottleneck associated with the invasion in Mediterranean Siganidae, and native and invasive populations are not genetically different [5, 6, 35]. This absence of reduced genetic diversity in the non-native populations could explain why microbiome diversity is high in the invasive populations. Further studies accounting for the genetic differences between individuals and populations are needed in order to fully appreciate how the microbiome is determined by the genetic background of the host.

A second explanation is that the microbial communities from the surrounding environment differ between the native and invaded ranges, as suggested by the significant differences between regions in the structure of sediment, turf and water microbiomes. Thus, as the first colonization of a fish gut by environmental microbes is known to influence the composition of its microbiome during the whole life of the host [3, 47], the influence of the environment cannot be ruled out. However, this initial microbial inoculate undergoes later modulations that depend on the fish interactions with environment, such as its foraging mode and range of food source. It is worth noting here that the microbiome of Siganidae was found to be distinct from the one of its putative dietary sources (algae, seagrass and turf, Additional file 1: Table S2 and S3), as reported for other herbivorous fishes [26, 55]. Furthermore, while the differences between regions in the environmental microbiomes could explain the modifications of the Siganidae gut microbiome they could not explain its homogenization. Indeed, in the Mediterranean Sea, the water and sediment microbiomes were not more homogeneous than in the native range while the algae and turf microbiomes actually exhibited a higher variability.

A third explaining factor would be a modification of the diet as the three studied regions differ greatly regarding potential food sources and the studied species have modified their diet in the Eastern Mediterranean compared with the Red Sea to match the available resources [9]. In the native range, *S. luridus* was shown to consume mainly brown macroalgae (i.e. 83% of the diet) while *S. rivulatus* feed on equal proportions on short brown and red algae (33%) with also some green algae (18%) [46]. However, macroalgae tend to be scarce in the sampled locations and *Siganus spp.* preferentially graze on the turf (i.e. epilithic algal matrix) that covers extensively the shallow reef flats (personal observations). None of the two species consumed seagrasses that are represented by small patches of *Halophila spp.* [46]. In the Levantine Sea, both macroalgae and seagrasses have been overgrazed by *Siganus spp.* and rocky substrates are covered by turf [65], which is the main food source of *S. rivulatus* [65]. Finally, in Northern Crete, the three types of food are abundant, with diverse sets of macroalgae, extensive seagrass meadows (i.e. *Posidonia*, *Cymodocea*) and copious amounts of turf (personal observations). Consequently, the contrasting food sources available to Siganidae in the three regions [4], and the wider diversity in Northern Crete compared with Northern Red Sea could be responsible for diet shifts that resulted in the observed microbiome modifications. Indeed, a more diversified diet in the invaded range could favor a higher alpha diversity of the gut microbiomes and lead to a more homogenous structure within and between species. The hypothesis of diet change in the invaded range is supported by the different and more homogeneous functional potential related to the metabolism of SCFA in the gut microbiome of fishes from the Mediterranean Sea.

A fourth factor driving the microbiome shift could be the differences in the fish assemblages co-occurring with Siganidae. Indeed, it was shown that inter-host dispersal constitutes an important factor shaping the diversity and composition of fish gut microbiome, potentially overwhelming individual host factors [14]. In their native range, the two studied species represent less than 1% of the fish communities and the latter are more diversified than in the Mediterranean Sea [43, 64], which ultimately increases the chance that their microbiome is influenced by the surrounding species through inter-host microbial dispersal. For instance, during sampling in the Red Sea, we observed *S. rivulatus* grazing on the turf covering the reef calcareous slab (< 40 cm deep) in mixed schools with several species of surgeonfish (Acanthuridae) and parrotfish (Scaridae). This configuration increased the likelihood of a Siganidae eating the feces of other species. In contrast, in the sampled regions of the Mediterranean Sea (Levantine Sea and Northern Crete), Siganidae represent a much higher proportion of less diverse fish assemblages [62, 77]. Consequently, we can expect the importance of intraspecific interactions in inter-host microbial dynamics to be higher in the non-native range and to result in a homogenization of the gut microbiome.

## Conclusion and perspectives

Non-native species are now common in most ecosystems, but not all of them become abundant or exert a sufficiently high negative impact to be called “invasive”. The outcome of introductions depends on whether the ecological traits of individuals allow successful survival and reproduction in the colonized environment that could differ from the native one. Here, we introduced a conceptual framework describing how ecological traits change both within and between species during the invasion process (or any other shift in environment, Fig. 1). This framework was applied to describe the modifications of the taxonomic, phylogenetic and functional structure of the gut microbiome in two Lessepsian herbivorous fishes (*S. rivulatus* and *S. luridus*) during their range expansion in the Mediterranean Sea (Levantine Sea and Northern Crete).

We found that the diversity of the microbiome increased as the fishes progressed within the invaded range and that both the dominant bacterial lineages and functions became increasingly different from the native ones. Overall, the structure of the microbiome became more homogeneous within and between species, i.e. between-individuals differences decreased, which corresponded to the conceptual scenario presented in the last row of Fig. 1. These taxonomic/phylogenetic modifications of the microbiome were associated with changes in its functional potential related to SCFA metabolism, which supports the hypothesis that the microbiome participated in the adaption of the host to the novel environment and trophic resources. It was previously shown that certain Siganidae are able to maintain the functioning of the gut microbiome despite feeding on a diversity of novel food sources (kelp, sargassum, seagrass, macrophytes) and fermenting them thanks to different bacterial taxa [41]. Such adaptation of the microbiome to novel dietary resources while still fulfilling nutritional requirements is likely a key factor for the success of invasive species [70]. There are several research directions to better understand the causes and consequences of the *Siganus* invasion in the Mediterranean Sea. First, it would be relevant to extend the number of populations surveyed to test whether *Siganus* populations at the western (Sicily) and northern (Aegean Sea) edges of the invasion front host microbiomes similar to those in the sites where they are already abundant for decades. Second, it would be revealing to extend assessment of bacterial diversity in fishes and their environment during winter to test whether colder water drives changes in the gut microbiome of the Mediterranean *Siganus* populations. Third, the phylogenetic diversity of the gut microbiome of Mediterranean Siganidae should be compared with those of the native herbivorous fishes, *Sarpa salpa* (Sparidae) and *Sparisoma cretense* (Scaridae). Fourth, for all these species, estimations of the realized microbiome functions (e.g. by identifying SCFA pathways using metatranscriptomic approaches) will allow measuring impact of microbiome on the host (e.g. growth rate) and impacts on ecosystems through nutrient recycling.

## Supplementary Information

**Additional file 1:** Supplementary tables and figures.

## Data Availability

All the data supporting the results of this manuscript will be archived on Dryad upon acceptance of the manuscript and the data DOI will be included at the end of the article (Data are currently hosted on the Dryad website and have been assigned the temporary https://doi.org/10.5061/dryad.j0zpc86fh). The codes used for analyses is available online: https://github.com/sebastienvilleger/exofishmed_microbiome_homogenization.
