# Supplementary material for: Shift and homogenization of gut microbiome during invasion in marine fishes
Source: Anim Microbiome. 2022 Jun 4;4:37. doi: 10.1186/s42523-022-00181-0 (PMC9167558; doi:10.1186/s42523-022-00181-0)
Supplement: Supplementary file 1 — Additional file 1: Supplementary tables and figures. [file 42523_2022_181_MOESM1_ESM.pdf]

Table S1: Summary of the sampling sites used in this study

Region	Sampling dates		Sampling sites			Habitat	Temperature (°C)		Salinity		pH		Chlorophyll-a ($mg.L^{-1}$)	
	**Spring**	**Autumn**	**Name**	**Longitude**	**Latitude**		**Spring**	**Autumn**	**Spring**	**Autumn**	**Spring**	**Autumn**	**Spring**	**Autumn**
**North Red Sea**														
	2018-06	2018-10	Eilat IUI	E 34.92238	N 29.49817	Coral reef	23-28	24-27	40.6-40.7	40.5-40.8	8.2-8.2	8.2-8.2	0.17-0.27	0.15-0.37
			Eilat north beach	E 34.96054	N 29.54178	Coral reef								
**Levantine Sea**														
	2018-06	2018-10	Mikhmoret	E 34.86620	N 32.4066	Sand and rock covered with turf	23-27	25-31	40-41	40-42	8.1-8.2	8.1-8.2	0.09-0.17	0.10-0.43
			Sdot Yam	E 34.887522	N 32.492817	Sand and rock covered with turf								
**Northern Crete**														
	2019-06	2019-10	Bali	E 24.808517	N 35.411617	Rock, sand and seagrass patches	22-26	22-23	40-41	40-41	8.1-8.2	8.1-8.2	0.10-0.15	0.10-0.20
			Hersonissos	E 25.386977	N 35.330012	Rock, sand and seagrass patches								
			Kokkini Hani	E 25.253880	N 35.332325	Rock, sand and seagrass patches								
			Psaromoura	E 25.019226	N 35.413666	Rock, sand and seagrass patches								

Table S2: Summary of the samples used in this study

Region	Sampling site	Fish				Turf		Macroalgae		Water		Sediment		Seagrass		Total	
		S. rivulatus		*S. luridus*													
		Spring	Autumn	Spring	Autumn	Spring	Autumn	Spring	Autumn	Spring	Autumn	Spring	Autumn	Spring	Autumn	**Spring**	**Autumn**
**North Red Sea**																	
	Eilat IUI	6	9	5	3	6	5			3	5	2	5				
	Eilat north beach	12	7	2		2	1			3	3	2	2				
	Total	*18*	*16*	*7*	*3*	*8*	*6*	*0*	*0*	*6*	*8*	*4*	*7*	*0*	*0*	***43***	***40***
**Levantine Sea**																	
	Mikhmoret	16	8			2											
	Sdot Yam	7	9			2	2	9	1		6						
	Total	*23*	*17*	*0*	*0*	*4*	*2*	*9*	*1*	*0*	*6*	*0*	*0*	*0*	*0*	***36***	***26***
**Northern Crete**																	
	Bali	4		5		3		6						3			
	Hersonissos	12	10	6	8		6	14	4	5	5	3	6	3	4		
	Kokkini Hani	10	15			3	7	17	5	5	2	7	4	6	8		
	Psaromoura				3		4				3				5		
	Total	*26*	*25*	*11*	*11*	*6*	*17*	*37*	*9*	*10*	*10*	*10*	*10*	*12*	*17*	***112***	***99***
**All three regions**																	
	**Total**	**67**	**58**	**18**	**14**	**18**	**25**	**46**	**10**	**16**	**24**	**14**	**17**	**12**	**17**	**191**	**165**
		**125**		**32**		**43**		**56**		**40**		**31**		**29**		**356**	

Table S3: Dominant phyla in ecosystem bacterial compartments

Relative abundance were computed on pooled samples. Values lower than 1% are not shown (-)

Phylum	Algae	Fish	Seagrass	Sediment	Turf	Water
Acidobacteria	-	-	-	2	-	-
Actinobacteria	3	-	5	3	2	4
Bacteroidetes	31	23	32	35	31	31
Calditrichaeota	-	-	-	-	-	-
Chloroflexi	1	-	2	1	-	-
Cyanobacteria	1	-	1	-	2	14
Dadabacteria	-	-	-	-	1	-
Deferribacteres	-	1	-	-	-	-
Deinococcus-Thermus	-	-	-	-	-	-
Epsilonbacteraeota	-	1	-	-	-	-
Firmicutes	-	27	2	-	1	-
Fusobacteria	-	3	-	-	-	-
Kiritimatiellaeota	-	-	-	-	-	-
Lentisphaerae	-	-	-	-	-	-
Other	-	-	-	-	-	-
Patescibacteria	-	-	1	-	-	-
Planctomycetes	2	-	4	6	4	-
Proteobacteria	55	32	50	50	57	49
Spirochaetes	-	1	-	1	-	-
Tenericutes	-	7	-	-	-	-
Verrucomicrobia	5	4	2	1	2	1

Table S4: Composition of the microbiome of different ecosystem compartments

Classes whose relative abundance were lower than 1% were depicted as "-".

Class	Algae	Fish	Seagrass	Sediment	Turf	Water
Acidimicrobiia	3	-	5	3	2	4
Alphaproteobacteria	40	1	36	13	41	37
Anaerolineae	1	-	2	1	-	-
Bacteroidia	31	23	32	34	31	29
Clostridia	-	21	2	-	1	-
Dadabacteriia	-	-	-	-	1	-
Deferribacteres	-	1	-	-	-	-
Deltaproteobacteria	1	31	3	11	3	1
Erysipelotrichia	-	7	-	-	-	-
Fusobacteriia	-	3	-	-	-	-
Gammaproteobacteria	14	1	12	25	13	11
Ignavibacteria	-	-	-	1	-	-
Mollicutes	-	7	-	-	-	-
Other	1	1	1	1	1	-
Oxyphotobacteria	1	-	1	-	2	14
Planctomycetacia	2	-	4	6	4	-
Rhodothermia	-	-	-	-	-	1
Saccharimonadia	-	-	-	-	-	-
Spirochaetia	-	-	-	1	-	-
Thermoanaerobaculia	-	-	-	2	-	-
Verrucomicrobiae	5	4	2	1	2	1

Table S5: Determinants of the structure of the microbiome in different ecosystem compartments

This table contains all the PERMANOVA tests performed using taxonomic dissimilarity indices, while taking or not into account relative abundances (*i.e.* q = 0 or 1, respectively) of taxa (Phyla, families or ASVs).

Ecosystem compartment	Rank	Diversity index	Factor	R^2	F-value	p-value		Ecosystem compartment	Rank	Diversity index	Factor	R^2	F-value	p-value	
algae	ASV	Taxonomic q0	Region	0.12	7.2	0.001	***	turf	ASV	Taxonomic q0	Region	0.219	5.9	0.001	***
algae	ASV	Taxonomic q0	Season	0.05	3.0	0.001	***	turf	ASV	Taxonomic q0	Season	0.049	2.7	0.002	**
algae	ASV	Taxonomic q0	Region:Season	0.02	1.4	0.060		turf	ASV	Taxonomic q0	Region:Season	0.078	2.2	0.001	***
algae	Family	Taxonomic q0	Region	0.14	8.9	0.001	***	turf	Family	Taxonomic q0	Region	0.289	8.6	0.001	***
algae	Family	Taxonomic q0	Season	0.05	3.3	0.001	***	turf	Family	Taxonomic q0	Season	0.052	3.1	0.004	**
algae	Family	Taxonomic q0	Region:Season	0.02	1.3	0.190		turf	Family	Taxonomic q0	Region:Season	0.078	2.5	0.001	**
algae	Phylum	Taxonomic q0	Region	0.03	1.7	0.181		turf	Phylum	Taxonomic q0	Region	0.349	11.0	0.001	***
algae	Phylum	Taxonomic q0	Season	0.08	4.5	0.002	**	turf	Phylum	Taxonomic q0	Season	0.008	0.5	0.672	
algae	Phylum	Taxonomic q0	Region:Season	0.01	0.5	0.713		turf	Phylum	Taxonomic q0	Region:Season	0.118	4.4	0.002	**
algae	ASV	Taxonomic q1	Region	0.16	10.5	0.001	***	turf	ASV	Taxonomic q1	Region	0.304	9.5	0.001	***
algae	ASV	Taxonomic q1	Season	0.06	4.0	0.001	***	turf	ASV	Taxonomic q1	Season	0.06	3.7	0.001	***
algae	ASV	Taxonomic q1	Region:Season	0.02	1.5	0.048	*	turf	ASV	Taxonomic q1	Region:Season	0.089	3.1	0.001	***
algae	Family	Taxonomic q1	Region	0.09	5.7	0.002	**	turf	Family	Taxonomic q1	Region	0.317	10.2	0.001	***
algae	Family	Taxonomic q1	Season	0.04	2.2	0.101		turf	Family	Taxonomic q1	Season	0.079	5.1	0.001	***
algae	Family	Taxonomic q1	Region:Season	0.01	0.4	0.608		turf	Family	Taxonomic q1	Region:Season	0.166	7.0	0.001	***
algae	Phylum	Taxonomic q1	Region	0.03	1.8	0.222		turf	Phylum	Taxonomic q1	Region	0.222	7.0	0.001	**
algae	Phylum	Taxonomic q1	Season	0.04	2.0	0.191		turf	Phylum	Taxonomic q1	Season	0.115	7.3	0.004	**
algae	Phylum	Taxonomic q1	Region:Season	-0.01	-0.5	0.984		turf	Phylum	Taxonomic q1	Region:Season	0.102	3.7	0.031	*
sediment	ASV	Taxonomic q0	Region	0.24	9.4	0.001	***	water	ASV	Taxonomic q0	Region	0.463	17.9	0.001	***
sediment	ASV	Taxonomic q0	Season	0.06	2.4	0.012	*	water	ASV	Taxonomic q0	Season	0.053	4.1	0.004	**
sediment	ASV	Taxonomic q0	Region:Season	0.04	1.7	0.055		water	ASV	Taxonomic q0	Region:Season	0.046	3.8	0.005	**
sediment	Family	Taxonomic q0	Region	0.39	21.1	0.001	***	water	Family	Taxonomic q0	Region	0.329	9.6	0.001	***
sediment	Family	Taxonomic q0	Season	0.06	3.1	0.018	*	water	Family	Taxonomic q0	Season	0.054	3.1	0.015	*
sediment	Family	Taxonomic q0	Region:Season	0.02	1.1	0.332		water	Family	Taxonomic q0	Region:Season	0.096	6.5	0.001	***
sediment	Phylum	Taxonomic q0	Region	0.28	11.6	0.001	***	water	Phylum	Taxonomic q0	Region	0.208	5.2	0.001	***
sediment	Phylum	Taxonomic q0	Season	0.05	1.9	0.121		water	Phylum	Taxonomic q0	Season	0.093	4.7	0.003	***
sediment	Phylum	Taxonomic q0	Region:Season	0.03	1.1	0.379		water	Phylum	Taxonomic q0	Region:Season	0.136	8.2	0.001	***
sediment	ASV	Taxonomic q1	Region	0.36	17.4	0.001	***	water	ASV	Taxonomic q1	Region	0.619	39.8	0.001	***
sediment	ASV	Taxonomic q1	Season	0.07	3.3	0.004	**	water	ASV	Taxonomic q1	Season	0.064	8.2	0.001	***
sediment	ASV	Taxonomic q1	Region:Season	0.04	2.2	0.039	*	water	ASV	Taxonomic q1	Region:Season	0.064	10.3	0.001	***
sediment	Family	Taxonomic q1	Region	0.54	52.8	0.001	***	water	Family	Taxonomic q1	Region	0.59	31.7	0.001	***
sediment	Family	Taxonomic q1	Season	0.17	16.2	0.001	***	water	Family	Taxonomic q1	Season	0.082	8.8	0.001	***
sediment	Family	Taxonomic q1	Region:Season	0.02	2.4	0.125		water	Family	Taxonomic q1	Region:Season	0.11	17.1	0.001	***
sediment	Phylum	Taxonomic q1	Region	0.46	23.9	0.001	***	water	Phylum	Taxonomic q1	Region	0.682	105.7	0.001	***
sediment	Phylum	Taxonomic q1	Season	0.02	1.1	0.345		water	Phylum	Taxonomic q1	Season	0.203	63.0	0.001	***
sediment	Phylum	Taxonomic q1	Region:Season	0.12	7.9	0.011	*	water	Phylum	Taxonomic q1	Region:Season	-0.019	-5.017	1	

Figure S1: Regional and seasonal differences in the structure of the microbiome in different ecosystem compartments

Principal Coordinates Analyses (PCoA) were performed on taxonomic dissimilarity (q = 1) estimated at the Family level. For each ecosystem compartment, red, blue and green dots correspond to Red Sea, Levantine Sea and Northern Crete, respectively. Filled and empty dots corresponds to spring and autumn, respectively. For both Siganidae species (the two bottom rows), the first two pairs of PCoA axes are represented (PC1-PC2 and PC3-PC4).

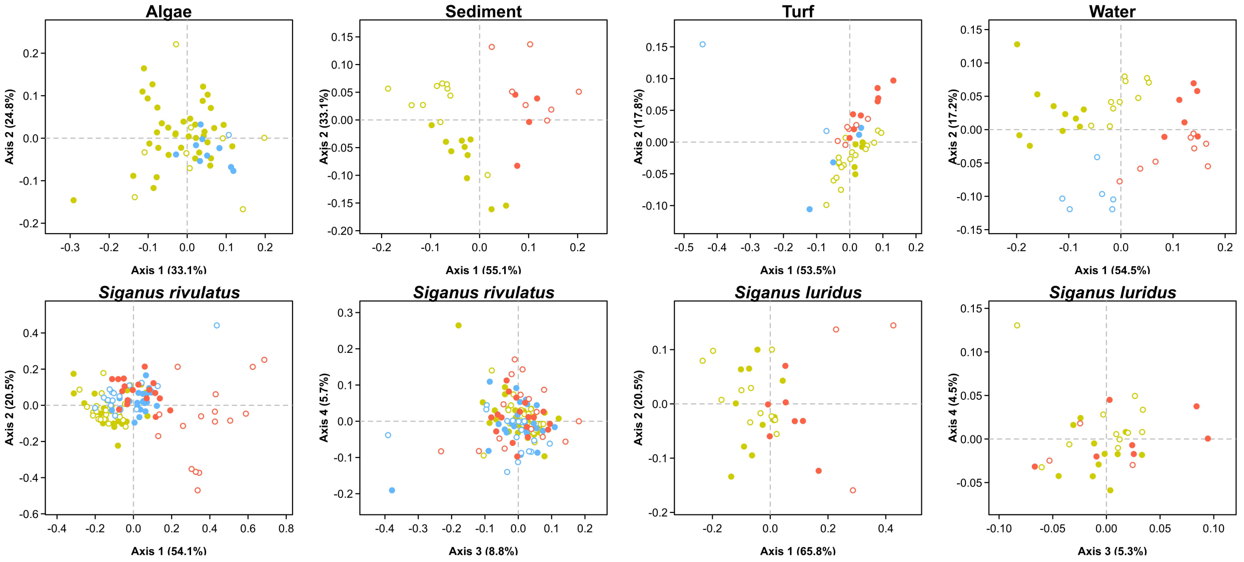

Figure S2: Alpha diversity in the microbiome of different ecosystem compartments
Diversity was estimated using the Hill' numbers framework for q = 1. Taxonomic and phylogenetic diversity for *S. rivulatus* and *S. luridus* was estimated on the core microbiome.

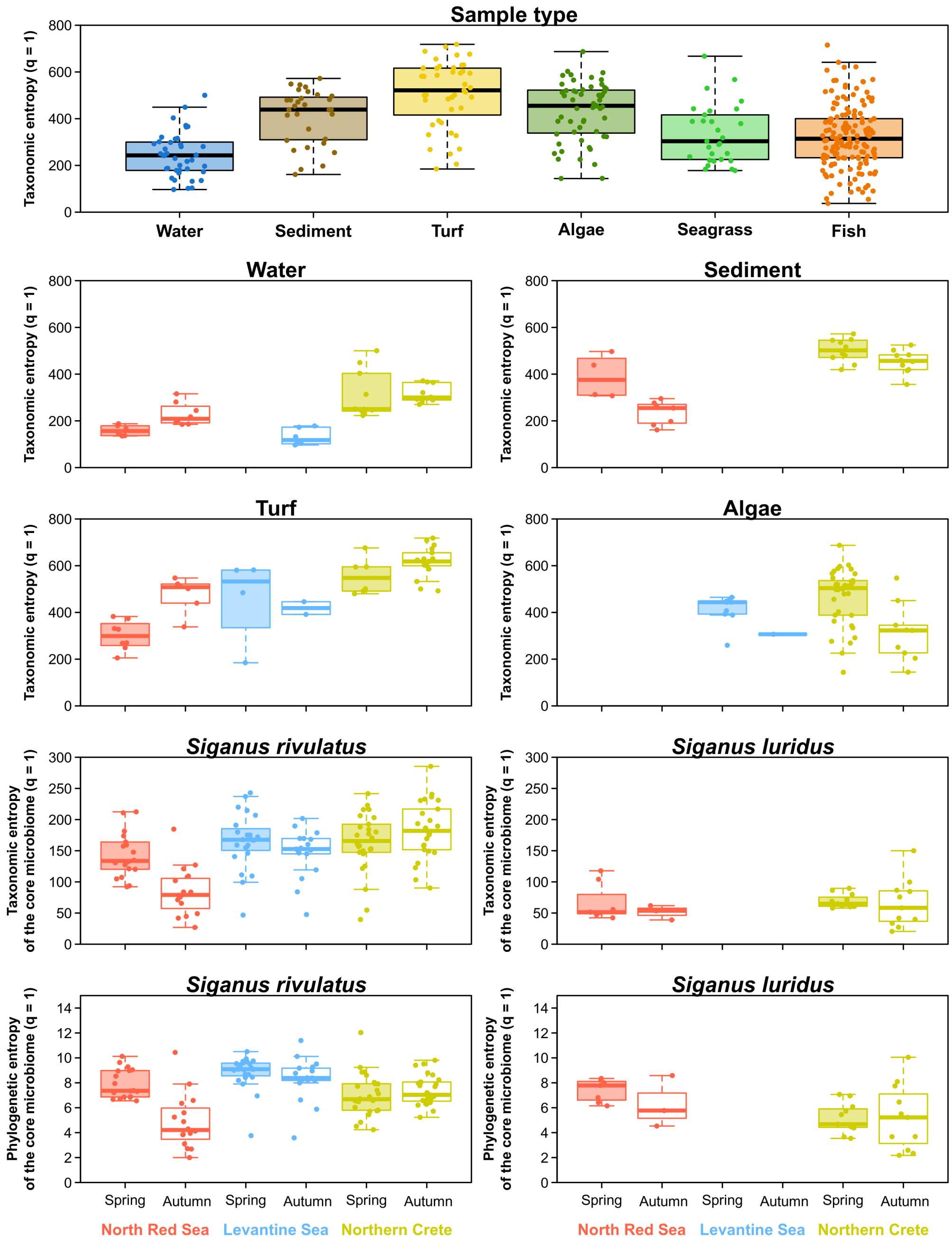

Table S6: Alpha diversity in the microbiome of different ecosystem compartments

Diversity was estimated using the Hill' numbers framework.

	Taxonomic ASVs richness					Taxonomic ASVs diversity (q= 1)				
**Sample type**	**Mean**	**±**	**SD**	**Min**	**Max**	**Mean**	**±**	**SD**	**Min**	**Max**
**Algae**	647	±	138	298	922	432	±	126	144	687
**Fish**	509	±	153	114	920	322	±	129	37	715
**Seagrass**	518	±	150	278	885	335	±	126	178	667
**Sediment**	615	±	130	328	773	411	±	117	161	572
**Turf**	724	±	152	403	943	506	±	142	185	718
**Water**	405	±	135	209	761	246	±	95	97	500

Table S7: Test of differences in alpha diversity between different ecosystem compartments

Kruskal Wallis test between sample types

Diversity index	# of significant test (n = 100)	Statistic		p-value	
		Mean	SD	Mean	SD
Taxonomic q0	100	61.4	6.7	0.000	0.000
Taxonomic q1	100	57.2	5.3	0.000	0.000

Dunn test between sample types

Diversity index	Pairwise comparison	# of significant test (n = 100)	Statistic		p-value	
			Mean	SD	Mean	SD
Taxonomic q0	Turf - Water	100	6.7	0.4	0.000	0.000
	Algae - Water	100	5.2	0.4	0.000	0.000
	Sediment - Water	100	4.5	0.3	0.000	0.000
	Algae - Seagrass	100	2.9	0.4	0.003	0.004
	Seagrass - Water	100	2.3	0.3	0.014	0.009
	Seagrass - Sediment	100	-2.2	0.2	0.014	0.005
	Seagrass - Turf	100	-4.5	0.3	0.000	0.000
	Fish - Turf	100	-4.6	0.6	0.000	0.000
	Algae - Fish	97	3.0	0.6	0.007	0.020
	Sediment - Turf	95	-2.2	0.4	0.018	0.016
	Fish - Sediment	86	-2.3	0.6	0.024	0.040
	Fish - Water	83	2.2	0.6	0.032	0.044
	Algae - Turf	40	-1.6	0.5	0.079	0.065
	Algae - Sediment	1	0.7	0.4	0.260	0.119
	Fish - Seagrass	0	-0.1	0.6	0.335	0.108
Taxonomic q1	Turf - Water	100	6.5	0.4	0.000	0.000
	Algae - Water	100	4.9	0.4	0.000	0.000
	Sediment - Water	100	4.4	0.3	0.000	0.000
	Algae - Seagrass	100	2.7	0.4	0.006	0.007
	Seagrass - Water	100	2.2	0.2	0.015	0.009
	Seagrass - Sediment	100	-2.1	0.2	0.017	0.007
	Seagrass - Turf	100	-4.3	0.4	0.000	0.000
	Fish - Turf	100	-4.4	0.6	0.000	0.000
	Algae - Fish	97	2.8	0.6	0.008	0.013
	Sediment - Turf	87	-2.2	0.5	0.025	0.025
	Fish - Sediment	87	-2.2	0.5	0.027	0.039
	Fish - Water	82	2.2	0.6	0.033	0.049
	Algae - Turf	45	-1.6	0.5	0.082	0.075
	Algae - Sediment	3	0.6	0.5	0.289	0.124
	Fish - Seagrass	0	-0.1	0.6	0.338	0.107

Table S8: Effect of region and season on the alpha diversity of different ecosystem compartments

ANOVA tests were performed only for the compartments for which we had at least two regions and two seasons. To account for differences in the number of samples for each region and season combinations, we used a bootstrap approach that consisted of running the ANOVA analysis one hundred time on randomly draw and equivalent number of samples.

ANOVA on alpha diversity for each sample type

Diversity index	Sample type	# of significant test (n = 100)	Factor	F-value		p-value		R2	
				Mean	SD	Mean	SD	Mean	SD
Taxonomic q0	**Sediment**	**100**	**region**	**26.3**	**5.7**	**0.000**	**0.000**	**0.70**	**0.05**
		**99**	**season**	**10.3**	**3.5**	**0.012**	**0.010**	**0.70**	**0.05**
		5	region:season	2.4	1.3	0.189	0.101	0.70	0.05
	**Turf**	**82**	**region**	**10.4**	**9.3**	**0.030**	**0.032**	**0.66**	**0.16**
		6	season	2.3	2.9	0.344	0.263	0.66	0.16
		46	region:season	7.4	9.8	0.126	0.140	0.66	0.16
	**Water**	**100**	**region**	**15.8**	**3.5**	**0.000**	**0.000**	**0.53**	**0.05**
		34	season	3.7	2.6	0.127	0.110	0.53	0.05
		9	region:season	2.5	1.3	0.177	0.145	0.53	0.05
Taxonomic q1	**Sediment**	**100**	**region**	**27.2**	**7.2**	**0.000**	**0.000**	**0.71**	**0.05**
		**97**	**season**	**10.0**	**3.1**	**0.013**	**0.014**	**0.71**	**0.05**
		22	region:season	3.2	1.9	0.157	0.127	0.71	0.05
	Turf	54	region	9.3	11.1	0.066	0.066	0.53	0.25
		12	season	3.6	6.6	0.275	0.232	0.53	0.25
		32	region:season	7.5	19.1	0.363	0.328	0.53	0.25
	**Water**	**100**	**region**	**25.4**	**5.6**	**0.000**	**0.000**	**0.64**	**0.05**
		30	season	4.4	5.3	0.179	0.192	0.64	0.05
		10	region:season	2.2	1.5	0.238	0.198	0.64	0.05

Figure S3: Composition of the core gut microbiome in Mediterranean Siganidae

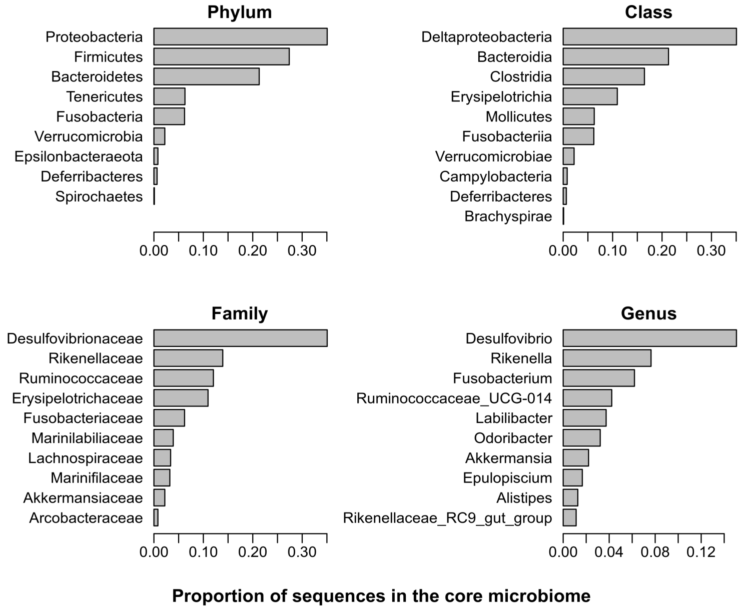

Identification of Siganidae core microbiome

The identification of the core gut microbiome of Siganidae from the three regions yielded contrasted patterns (Figure S4 and S5). For both species, the core microbiome was well identified in the native range, with the large majority of ASVs being considered as members of the core in > 80% of the 1000 bootstrapped iterations. On the contrary, in the two non-native populations, the core microbiome was loosely identified as many ASVs were identified as core only in few iterations, which resulted in a multimodal distribution of the number of time each ASV was member of the core. For both species, the core microbiome represented a higher proportion of the total number of sequences in the native range compared with the two non-native regions (Figure S6), but the differences was significant only in *S. rivulatus* (Kruskall-Wallis, p-value < 0.001, Table S8 and S9).

Figure S4: Results of the core identification algorithm for *Siganus rivulatus*

Distributions were generated from 1000 iterations with random subsamples of similar numbers of individuals for each region (n = 14). Top plots represent the number of ASVs identified as core in the 1000 iterations and bottom plots represent the number of iterations in which each ASV is considered as part of the core.

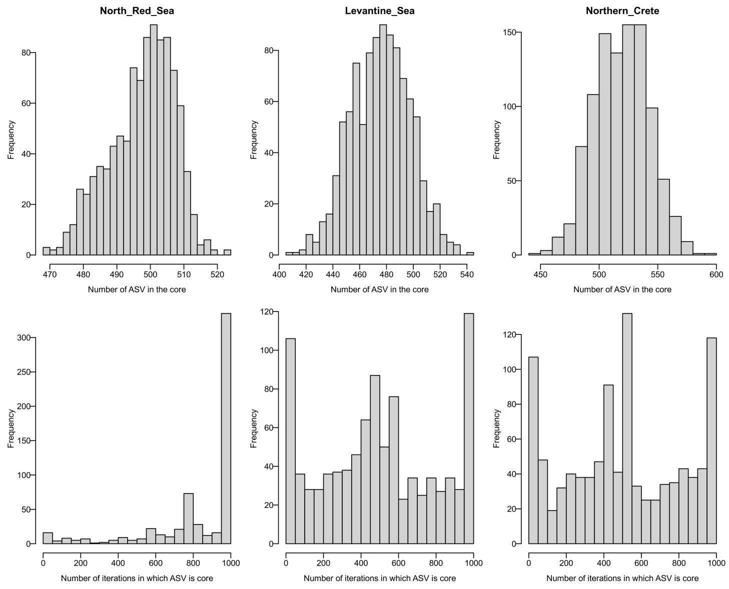

Figure S5: Results of the core identification algorithm for *Siganus luridus*

Distributions were generated from 1000 iterations with random subsamples of similar numbers of individuals for each region (n = 3).

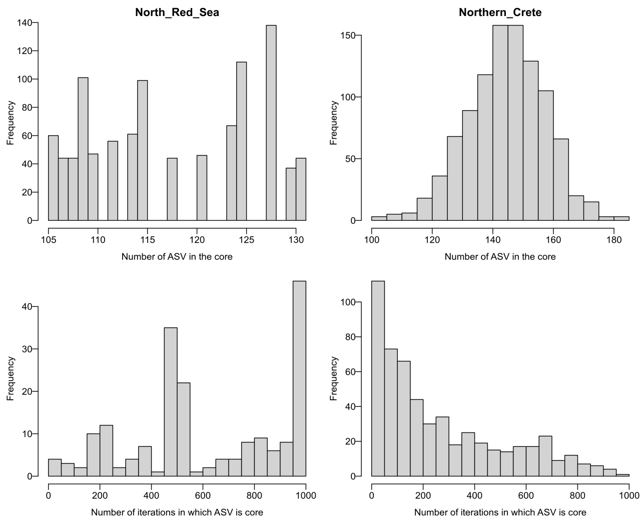

Figure S6: Contribution of the core microbiome to the total microbiome in Siganidae from different regions

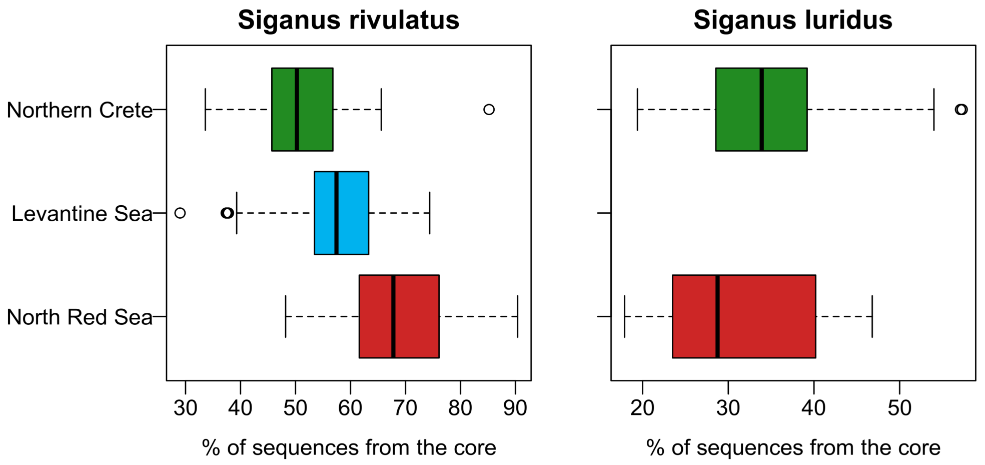

Table S9: Differences in the proportion of the total gut microbiome represented by the core microbiome in different regions

Statistic and p-values correspond to Kruskall-Wallis rank sum test.

Species	Response variable	Statistic	p-value	
Siganus rivulatus	% of sequences from the core	47.2	0.000	***
Siganus rivulatus	% of ASVs from the core	41.5	0.000	***
Siganus luridus	% of sequences from the core	1.0	0.330	
Siganus luridus	% of ASVs from the core	7.0	0.008	**

Table S10: Pairwise differences in the proportion of the total gut microbiome represented by the core microbiome in different regions

Statistic and p-values correspond to Dunn test.

Species	Response variable	Pairwise comparison	Statistic	p-value	
Siganus rivulatus	% of sequences from the core	Levantine Sea - North Red Sea	-3.8	0.000	***
Siganus rivulatus	% of sequences from the core	Levantine Sea - Northern Crete	3.5	0.000	***
Siganus rivulatus	% of sequences from the core	North Red Sea - Northern Crete	6.9	0.000	***
Siganus rivulatus	% of ASVs from the core	Levantine Sea - North Red Sea	2.1	0.016	*
Siganus rivulatus	% of ASVs from the core	Levantine Sea - Northern Crete	-4.5	0.000	***
Siganus rivulatus	% of ASVs from the core	North Red Sea - Northern Crete	-6.1	0.000	***
Siganus luridus	% of sequences from the core	North Red Sea - Northern Crete	-1.0	0.165	
Siganus luridus	% of ASVs from the core	North Red Sea - Northern Crete	-2.7	0.004	**

Table S11: Composition of the microbiome of *S. rivulatus* and *S. luridus* in their native and invaded ranges

Phyla whose relative abundance were lower than 1% were depicted as "-".

	North Red Sea		Levantine Sea	Northern Crete	
**Phylum**	*S. rivulatus*	*S. luridus*	*S. rivulatus*	*S. rivulatus*	*S. luridus*
Bacteroidetes	15	19	26	25	7
Deferribacteres	-	-	1	1	1
Epsilonbacteraeota	-	-	-	2	1
Firmicutes	48	36	29	12	12
Fusobacteria	6	5	5	6	6
Proteobacteria	17	30	31	47	66
Spirochaetes	-	-	-	-	-
Tenericutes	13	9	4	3	6
Verrucomicrobia	1	-	4	3	1

Table S12: Composition of the microbiome of *S. rivulatus* and *S. luridus* in their native and invaded ranges

Families whose relative abundance were lower than 1% were depicted as "-".

				North Red Sea		Levantine Sea	Northern Crete	
**Phylum**	**Class**	**Order**	**Family**	*S. rivulatus*	*S. luridus*	*S. rivulatus*	*S. rivulatus*	*S. luridus*
Bacteroidetes	Bacteroidia	Bacteroidales	Marinifilaceae	3	9	4	3	4
Bacteroidetes	Bacteroidia	Bacteroidales	Marinilabiliaceae	2	1	4	8	0
Bacteroidetes	Bacteroidia	Bacteroidales	Rikenellaceae	12	11	18	16	4
Bacteroidetes	Bacteroidia	Bacteroidales	Rs-E47_termite_group	-	-	-	-	-
Bacteroidetes	Bacteroidia	Bacteroidales	Tannerellaceae	-	-	1	-	-
Deferribacteres	Deferribacteres	Deferribacterales	Deferribacteraceae	-	-	1	1	1
Epsilonbacteraeota	Campylobacteria	Campylobacterales	Arcobacteraceae	-	-	-	2	1
Firmicutes	Clostridia	Clostridiales	Christensenellaceae	-	-	-	-	-
Firmicutes	Clostridia	Clostridiales	Family_XI	1	-	-	-	-
Firmicutes	Clostridia	Clostridiales	Family_XIII	1	1	1	-	-
Firmicutes	Clostridia	Clostridiales	Lachnospiraceae	4	4	7	1	1
Firmicutes	Clostridia	Clostridiales	Ruminococcaceae	25	33	9	8	10
Firmicutes	Erysipelotrichia	Erysipelotrichales	Erysipelotrichaceae	25	3	13	2	2
Fusobacteria	Fusobacteriia	Fusobacteriales	Fusobacteriaceae	7	6	5	6	7
Proteobacteria	Alphaproteobacteria	Rhodobacterales	Rhodobacteraceae	-	-	-	-	-
Proteobacteria	Deltaproteobacteria	Desulfovibrionales	Desulfovibrionaceae	19	33	32	49	71
Spirochaetes	Brachyspirae	Brachyspirales	Brachyspiraceae	-	-	-	-	-
Tenericutes	Mollicutes	Anaeroplasmatales	Anaeroplasmataceae	-	-	-	-	-
Verrucomicrobia	Verrucomicrobiae	Verrucomicrobiales	Akkermansiaceae	1	-	4	3	1

Table S13: The two Mediterranean Siganidae host a different microbiome

This table contains the p-values the PERMANOVA testing the effect of species on the microbiome composition and structure. These tests were performed using taxonomic and phylogenetic dissimilarity indices, while taking or not into account relative abundances (*i.e.* q = 0 or 1, respectively) of taxa (Phyla, classes, families or ASVs).

North Red Sea				Northern Crete			
**Rank**	**Diversity index**	**p.value**		**Rank**	**Diversity index**	**p.value**	
Phylum	Taxonomic q0	0.201		Phylum	Taxonomic q0	0.001	***
	Taxonomic q1	0.857			Taxonomic q1	0.001	***
	Phylogenetic q0	0.206			Phylogenetic q0	0.001	***
	Phylogenetic q1	0.845			Phylogenetic q1	0.001	***
Class	Taxonomic q0	0.185		Class	Taxonomic q0	0.001	***
	Taxonomic q1	0.1			Taxonomic q1	0.001	***
	Phylogenetic q0	0.166			Phylogenetic q0	0.001	***
	Phylogenetic q1	0.173			Phylogenetic q1	0.001	***
Family	Taxonomic q0	0.003	**	Family	Taxonomic q0	0.001	***
	Taxonomic q1	0.01	**		Taxonomic q1	0.001	***
	Phylogenetic q0	0.01	**		Phylogenetic q0	0.001	***
	Phylogenetic q1	0.026	*		Phylogenetic q1	0.001	***
ASV	Taxonomic q0	0.001	**	ASV	Taxonomic q0	0.001	***
	Taxonomic q1	0.001	*		Taxonomic q1	0.001	***
	Phylogenetic q0	0.001	***		Phylogenetic q0	0.001	***
	Phylogenetic q1	0.013	*		Phylogenetic q1	0.001	***

Table S14: There is no intra region differences in the composition of the gut microbiome

This table summarizes the results of the PERMANOVA testing for differences between sites within each region. These tests were performed using taxonomic and phylogenetic dissimilarity indices, while taking or not into account relative abundances (*i.e.* q = 0 or 1, respectively) of taxa (Phyla, families or ASVs). The total number of test for each species-region-season combination is equal to 12 (*i.e.* two types of diversity times two values of q times three levels of taxonomic resolution).

Species	Region	Season	Average p-value	Average F-value	Average R^2	# of significant tests
Siganus luridus	North Red Sea	Autumn	0.511	0.89	0.09	0
		Spring	0.509	0.89	0.09	0
	Northern Crete	Autumn	0.369	1.16	0.11	0
		Spring	0.361	1.16	0.11	0
Siganus rivulatus	Levantine Sea	Autumn	0.349	1.35	0.03	0
		Spring	0.354	1.35	0.03	0
	North Red Sea	Autumn	0.318	1.18	0.04	0
		Spring	0.333	1.18	0.04	0
	Northern Crete	Autumn	0.220	1.52	0.06	0
		Spring	0.228	1.52	0.06	0

Table S15: Determinants of the structure of the microbiome in Siganidae

This table contains all the PERMANOVA tests performed using taxonomic and phylogenetic dissimilarity indices, while taking or not into account relative abundances (*i.e.* q = 0 or 1, respectively) of taxa (Phyla, families or ASVs).

Ecosystem compartment	Rank	Diversity index	Factor	R^2	F-value	p-value		Ecosystem compartment	Rank	Diversity index	Factor	R^2	F-value	p-value	
S. luridus	ASV	Phylogenetic q0	Region	0.12	3.9	0.003	**	*S. rivulatus*	ASV	Phylogenetic q0	Region	0.233	18.5	0.001	***
S. luridus	ASV	Phylogenetic q0	Season	0.06	1.8	0.085		*S. rivulatus*	ASV	Phylogenetic q0	Season	0.032	4.1	0.003	**
S. luridus	ASV	Phylogenetic q0	Region:Season	0.06	2.2	0.039	*	*S. rivulatus*	ASV	Phylogenetic q0	Region:Season	0.099	9.3	0.001	***
S. luridus	Family	Phylogenetic q0	Region	0.23	8.7	0.001	***	*S. rivulatus*	Family	Phylogenetic q0	Region	0.256	21.0	0.001	***
S. luridus	Family	Phylogenetic q0	Season	0.08	2.7	0.07		*S. rivulatus*	Family	Phylogenetic q0	Season	0.041	5.3	0.002	**
S. luridus	Family	Phylogenetic q0	Region:Season	0.14	6.9	0.001	**	*S. rivulatus*	Family	Phylogenetic q0	Region:Season	0.114	11.6	0.001	***
S. luridus	Phylum	Phylogenetic q0	Region	0.39	19.1	0.001	***	*S. rivulatus*	Phylum	Phylogenetic q0	Region	0.305	26.8	0.001	***
S. luridus	Phylum	Phylogenetic q0	Season	0.06	2.0	0.17		*S. rivulatus*	Phylum	Phylogenetic q0	Season	0.053	6.9	0.009	**
S. luridus	Phylum	Phylogenetic q0	Region:Season	0.15	9.2	0.002	**	*S. rivulatus*	Phylum	Phylogenetic q0	Region:Season	0.141	16.9	0.001	***
S. luridus	ASV	Phylogenetic q1	Region	0.41	21.0	0.001	***	*S. rivulatus*	ASV	Phylogenetic q1	Region	0.422	44.5	0.001	***
S. luridus	ASV	Phylogenetic q1	Season	0.02	0.5	0.563		*S. rivulatus*	ASV	Phylogenetic q1	Season	0.006	0.7	0.456	
S. luridus	ASV	Phylogenetic q1	Region:Season	0.16	11.3	0.003	**	*S. rivulatus*	ASV	Phylogenetic q1	Region:Season	0.247	46.2	0.001	***
S. luridus	Family	Phylogenetic q1	Region	0.55	36.1	0.001	***	*S. rivulatus*	Family	Phylogenetic q1	Region	0.461	52.2	0.001	***
S. luridus	Family	Phylogenetic q1	Season	-0.03	-0.8	0.947		*S. rivulatus*	Family	Phylogenetic q1	Season	-0.03	-3.37	1.00	
S. luridus	Family	Phylogenetic q1	Region:Season	0.19	19.4	0.001	***	*S. rivulatus*	Family	Phylogenetic q1	Region:Season	0.283	60.9	0.001	***
S. luridus	Phylum	Phylogenetic q1	Region	0.58	40.9	0.001	***	*S. rivulatus*	Phylum	Phylogenetic q1	Region	0.482	56.8	0.001	***
S. luridus	Phylum	Phylogenetic q1	Season	-0.05	-1.30	0.97		*S. rivulatus*	Phylum	Phylogenetic q1	Season	-0.03	-4.01	1.00	
S. luridus	Phylum	Phylogenetic q1	Region:Season	0.20	21.9	0.002	**	*S. rivulatus*	Phylum	Phylogenetic q1	Region:Season	0.338	99.3	0.001	***
S. luridus	ASV	Taxonomic q0	Region	0.12	4.1	0.002	**	*S. rivulatus*	ASV	Taxonomic q0	Region	0.212	16.4	0.001	***
S. luridus	ASV	Taxonomic q0	Season	0.05	1.6	0.092		*S. rivulatus*	ASV	Taxonomic q0	Season	0.029	3.7	0.002	**
S. luridus	ASV	Taxonomic q0	Region:Season	0.06	2.1	0.036	*	*S. rivulatus*	ASV	Taxonomic q0	Region:Season	0.101	9.1	0.001	***
S. luridus	Family	Taxonomic q0	Region	0.18	6.7	0.001	***	*S. rivulatus*	Family	Taxonomic q0	Region	0.238	19.0	0.001	***
S. luridus	Family	Taxonomic q0	Season	0.08	2.7	0.059		*S. rivulatus*	Family	Taxonomic q0	Season	0.038	4.8	0.003	**
S. luridus	Family	Taxonomic q0	Region:Season	0.12	5.3	0.003	**	*S. rivulatus*	Family	Taxonomic q0	Region:Season	0.099	9.5	0.001	***
S. luridus	Phylum	Taxonomic q0	Region	0.40	19.9	0.001	***	*S. rivulatus*	Phylum	Taxonomic q0	Region	0.297	25.8	0.001	***
S. luridus	Phylum	Taxonomic q0	Season	0.06	1.8	0.207		*S. rivulatus*	Phylum	Taxonomic q0	Season	0.051	6.6	0.002	**
S. luridus	Phylum	Taxonomic q0	Region:Season	0.15	9.5	0.005	**	*S. rivulatus*	Phylum	Taxonomic q0	Region:Season	0.136	15.8	0.001	***
S. luridus	ASV	Taxonomic q1	Region	0.18	6.7	0.001	***	*S. rivulatus*	ASV	Taxonomic q1	Region	0.255	20.9	0.001	***
S. luridus	ASV	Taxonomic q1	Season	0.08	2.5	0.032	*	*S. rivulatus*	ASV	Taxonomic q1	Season	0.037	4.7	0.002	**
S. luridus	ASV	Taxonomic q1	Region:Season	0.07	2.8	0.019	*	*S. rivulatus*	ASV	Taxonomic q1	Region:Season	0.125	12.8	0.001	***
S. luridus	Family	Taxonomic q1	Region	0.51	31.7	0.001	***	*S. rivulatus*	Family	Taxonomic q1	Region	0.421	44.4	0.001	***
S. luridus	Family	Taxonomic q1	Season	-0.01	-0.4	0.885		*S. rivulatus*	Family	Taxonomic q1	Season	-0.02	-1.97	1.00	
S. luridus	Family	Taxonomic q1	Region:Season	0.17	16.0	0.001	***	*S. rivulatus*	Family	Taxonomic q1	Region:Season	0.249	43.5	0.001	***
S. luridus	Phylum	Taxonomic q1	Region	0.57	40.3	0.001	***	*S. rivulatus*	Phylum	Taxonomic q1	Region	0.475	55.3	0.001	***
S. luridus	Phylum	Taxonomic q1	Season	-0.04	-1.20	0.98		*S. rivulatus*	Phylum	Taxonomic q1	Season	-0.03	-3.52	1.00	
S. luridus	Phylum	Taxonomic q1	Region:Season	0.20	21.4	0.001	**	*S. rivulatus*	Phylum	Taxonomic q1	Region:Season	0.335	96.0	0.001	***

Table S16: Pairwise comparisons of the microbiome structure in Siganidae

This table summarizes the results of the PERMANOVA testing for pairwise differences between regions. These tests were performed using taxonomic and phylogenetic dissimilarity indices, while taking or not into account relative abundances (*i.e.* q = 0 or 1, respectively) of taxa (Phyla, families or ASVs). The total number of test for each comparison is equal to 12 (*i.e.* two types of diversity times two values of q times three levels of taxonomic resolution).

Species	Comparison	# of significant tests	average F-value	average p-value	average R2
S. luridus	Northern_Crete_vs_North_Red_Sea	12	19.4	0.001	0.35
S. rivulatus	Levantine_Sea_vs_North_Red_Sea	12	16.7	0.001	0.18
	Northern_Crete_vs_Levantine_Sea	12	24.7	0.001	0.21
	Northern_Crete_vs_North_Red_Sea	12	64.4	0.001	0.38

Table S17: Alpha diversity in the core microbiome of Mediterranean Siganidae

Diversity was estimated using the Hill' numbers framework.

		Taxonomic richness			Taxonomic diversity			Phylogenetic richness			Phylogenetic diversity		
**Species**	**Region**	**Mean ± SD**	**Min**	**Max**	**Mean ± SD**	**Min**	**Max**	**Mean ± SD**	**Min**	**Max**	**Mean ± SD**	**Min**	**Max**
Siganus rivulatus													
	North Red Sea	204 ± 66	70	361	115 ± 47	27	212	31 ± 9	13	49	6 ± 2	2	10
	Levantine Sea	243 ± 64	71	380	157 ± 43	47	243	38 ± 8	13	55	9 ± 2	4	11
	Northern Crete	290 ± 55	95	393	172 ± 48	40	285	43 ± 7	17	54	7 ± 1	4	12
Siganus luridus													
	North Red Sea	116 ± 47	75	204	62 ± 27	39	118	22 ± 6	17	34	7 ± 1	5	9
	Northern Crete	135 ± 32	103	251	67 ± 28	21	150	24 ± 5	19	41	5 ± 2	2	10

			Taxonomic richness			Taxonomic diversity			Phylogenetic richness			Phylogenetic diversity		
**Species**	**Region**	**Season**	**Mean ± SD**	**Min**	**Max**	**Mean ± SD**	**Min**	**Max**	**Mean ± SD**	**Min**	**Max**	**Mean ± SD**	**Min**	**Max**
Siganus rivulatus														
	North Red Sea	Autumn	159 ± 53	70	266	84 ± 39	27	185	24 ± 8	13	44	5 ± 2	2	10
		Spring	243 ± 49	179	361	143 ± 36	92	212	38 ± 5	30	49	8 ± 1	7	10
	Levantine Sea	Autumn	218 ± 53	71	317	147 ± 39	48	202	36 ± 7	13	47	8 ± 2	4	11
		Spring	262 ± 66	117	380	165 ± 45	47	243	40 ± 8	22	55	9 ± 1	4	11
	Northern Crete	Autumn	307 ± 42	208	393	183 ± 47	90	285	45 ± 5	32	54	7 ± 1	5	10
		Spring	275 ± 62	95	356	163 ± 48	40	242	41 ± 8	17	51	7 ± 2	4	12
Siganus luridus														
	North Red Sea	Autumn	86 ± 3	82	88	51 ± 12	39	62	19 ± 2	18	21	6 ± 2	5	9
		Spring	128 ± 52	75	204	67 ± 31	42	118	24 ± 6	17	34	7 ± 1	6	8
	Northern Crete	Autumn	144 ± 42	103	251	65 ± 39	21	150	26 ± 6	19	41	5 ± 3	2	10
		Spring	125 ± 16	103	159	69 ± 11	58	90	22 ± 3	19	29	5 ± 1	4	7

Table S18: Effect of region and season on the alpha diversity of the core microbiome of Mediterranean Siganidae

To account for differences in the number of samples for each region and season combinations, we used a bootstrap approach that consisted of running the ANOVA analysis one hundred time on randomly draw and equivalent number of samples.

ANOVA on alpha diversity for each species

**Species**	**Diversity index**	**# of significant test (n = 100)**	**Factor**	**F-value Mean**	**F-value SD**	**p-value Mean**	**p-value SD**
Siganus rivulatus	Phylogenetic q0	100	Region	27.4	6.8	0.000	0.000
		100	Season	12.4	3.7	0.002	0.002
		100	Region:Season	13.6	2.2	0.000	0.000
	Phylogenetic q1	100	Region	16.0	3.0	0.000	0.000
		100	Season	11.1	2.6	0.002	0.003
		100	Region:Season	10.0	2.0	0.000	0.000
	Taxonomic q0	100	Region	22.5	4.6	0.000	0.000
		99	Season	8.7	3.2	0.011	0.014
		100	Region:Season	9.7	1.8	0.000	0.001
	Taxonomic q1	100	Region	16.7	3.7	0.000	0.000
		70	Season	5.2	2.3	0.052	0.066
		100	Region:Season	7.2	2.0	0.004	0.005
Siganus luridus	Phylogenetic q0	19	Region	3.2	4.8	0.349	0.279
		1	Season	0.8	1.0	0.555	0.273
		22	Region:Season	3.7	3.1	0.184	0.190
	Phylogenetic q1	21	Region	3.7	3.3	0.206	0.207
		2	Season	0.9	1.2	0.542	0.270
		2	Region:Season	0.7	1.1	0.563	0.254
	Taxonomic q0	30	Region	7.6	17.2	0.284	0.273
		5	Season	1.4	2.0	0.438	0.265
		17	Region:Season	3.5	3.2	0.192	0.182
	Taxonomic q1	4	Region	1.5	3.5	0.533	0.288
		7	Season	1.7	2.8	0.442	0.286
		5	Region:Season	1.4	3.7	0.558	0.277

Table S19: There is no intra region differences in the microbiome alpha diversity

To account for differences in the number of samples for each site within regions, we used a bootstrap approach that consisted of running the statistical tests (Wilcoxon when two sites were sampled and Kruskall-Wallis when more than two sites were sampled) one hundred time on randomly draw and equivalent number of samples. Here we report the average results across these 100 tests.

Species	Region	Diversity index	# of significant test (n = 100)	Test statistic		p-value	
				Mean	SD	Mean	SD
Siganus luridus	North Red Sea	Phylogenetic q0	0	0.0	0.0	0.333	0.000
		Phylogenetic q1	0	1.5	0.8	0.760	0.212
		Taxonomic q0	0	0.0	0.0	0.333	0.000
		Taxonomic q1	0	0.0	0.0	0.333	0.000
	Northern Crete	Phylogenetic q0	8	4.8	1.1	0.106	0.069
		Phylogenetic q1	16	5.0	1.3	0.104	0.077
		Taxonomic q0	15	5.2	0.8	0.082	0.032
		Taxonomic q1	20	5.5	0.8	0.069	0.025
Siganus rivulatus	Levantine Sea	Phylogenetic q0	0	117.7	11.1	0.683	0.223
		Phylogenetic q1	0	140.3	13.5	0.614	0.238
		Taxonomic q0	0	119.3	10.7	0.701	0.204
		Taxonomic q1	0	136.5	11.4	0.692	0.210
	North Red Sea	Phylogenetic q0	3	79.9	7.9	0.207	0.111
		Phylogenetic q1	56	62.7	8.4	0.051	0.036
		Taxonomic q0	0	89.9	9.3	0.394	0.191
		Taxonomic q1	12	73.4	6.9	0.125	0.070
	Northern Crete	Phylogenetic q0	2	2.9	1.4	0.286	0.160
		Phylogenetic q1	0	0.9	1.0	0.710	0.238
		Taxonomic q0	3	2.5	1.3	0.353	0.195
		Taxonomic q1	2	2.6	1.6	0.346	0.208

Table S20: Differentially abundant taxa between regions in Mediterranean Siganidae

This table contains the results of the Kruskal-Wallis tests comparing the CLR transformed abundances of bacterial taxa at various level of resolution (Phylum, Family, Genus) between different regions.

					Siganus rivulatus			***Siganus luridus***		
**Rank**	**Phylum**	**Class**	**Family**	**Genus**	**Statistic**	**p-value corrected**		**Statistic**	**p-value corrected**	
Phylum	Bacteroidetes				10	0.010	**	17	0.011	*
Phylum	Deferribacteres				45	0.000	***	1	0.389	
Phylum	Epsilonbacteraeota				30	0.000	***	5	0.080	
Phylum	Firmicutes				69	0.000	***	14	0.011	*
Phylum	Fusobacteria				15	0.001	**	1	0.347	
Phylum	Proteobacteria				1	0.521		2	0.259	
Phylum	Spirochaetes				8	0.023	*	5	0.080	
Phylum	Tenericutes				28	0.000	***	6	0.071	
Phylum	Verrucomicrobia				40	0.000	***	4	0.104	
Family	Bacteroidetes	Bacteroidia	Marinifilaceae		4	0.135		1	0.410	
Family	Bacteroidetes	Bacteroidia	Marinilabiliaceae		39	0.000	***	0	0.543	
Family	Bacteroidetes	Bacteroidia	Rikenellaceae		3	0.212		13	0.011	**
Family	Bacteroidetes	Bacteroidia	Rs-E47 termite group		14	0.002	**	0	0.837	
Family	Bacteroidetes	Bacteroidia	Tannerellaceae		32	0.000	***	0	0.837	
Family	Deferribacteres	Deferribacteres	Deferribacteraceae		55	0.000	***	8	0.038	*
Family	Epsilonbacteraeota	Campylobacteria	Arcobacteraceae		40	0.000	***	7	0.038	*
Family	Firmicutes	Clostridia	Christensenellaceae		19	0.000	***	0	0.971	
Family	Firmicutes	Clostridia	Family XI		2	0.328		1	0.474	
Family	Firmicutes	Clostridia	Family XIII		16	0.001	***	0	0.699	
Family	Firmicutes	Clostridia	Lachnospiraceae		30	0.000	***	13	0.011	*
Family	Firmicutes	Clostridia	Ruminococcaceae		20	0.000	***	8	0.038	
Family	Firmicutes	Erysipelotrichia	Erysipelotrichaceae		65	0.000	***	1	0.367	
Family	Fusobacteria	Fusobacteriia	Fusobacteriaceae		5	0.080		0	0.917	
Family	Proteobacteria	Alphaproteobacteria	Rhodobacteraceae		28	0.000	***	0	0.837	
Family	Proteobacteria	Deltaproteobacteria	Desulfovibrionaceae		34	0.000	***	11	0.024	*
Family	Spirochaetes	Brachyspirae	Brachyspiraceae		23	0.000	***	0	0.837	
Family	Tenericutes	Mollicutes	Anaeroplasmataceae		30	0.000	***	1	0.450	
Family	Verrucomicrobia	Verrucomicrobiae	Akkermansiaceae		41	0.000	***	8	0.038	
Genus	Bacteroidetes	Bacteroidia	Marinifilaceae	Odoribacter	4	0.152		0	0.917	
Genus	Bacteroidetes	Bacteroidia	Marinilabiliaceae	Labilibacter	49	0.000	***	0	0.758	
Genus	Bacteroidetes	Bacteroidia	Rikenellaceae	Alistipes	37	0.000	***	5	0.077	
Genus	Bacteroidetes	Bacteroidia	Rikenellaceae	dgA-11 gut group	23	0.000	***	0	0.647	
Genus	Bacteroidetes	Bacteroidia	Rikenellaceae	Rikenella	2	0.394		2	0.243	
Genus	Bacteroidetes	Bacteroidia	Rikenellaceae	Rikenellaceae RC9 gut group	23	0.000	***	4	0.104	
Genus	Bacteroidetes	Bacteroidia	Tannerellaceae	Macellibacteroides	29	0.000	***	2	0.200	
Genus	Epsilonbacteraeota	Campylobacteria	Arcobacteraceae	Arcobacter	44	0.000	***	9	0.038	*
Genus	Firmicutes	Clostridia	Christensenellaceae	Christensenella	11	0.006	**	1	0.518	
Genus	Firmicutes	Clostridia	Family XI	Sedimentibacter	3	0.276		0	0.813	
Genus	Firmicutes	Clostridia	Lachnospiraceae	Bacteroides	14	0.001	***	0	0.868	
Genus	Firmicutes	Clostridia	Lachnospiraceae	Epulopiscium	55	0.000	***	2	0.200	
Genus	Firmicutes	Clostridia	Lachnospiraceae	Faecalicatena	12	0.003	**	2	0.229	
Genus	Firmicutes	Clostridia	Lachnospiraceae	Tyzzerella	8	0.022	*	0	0.647	
Genus	Firmicutes	Clostridia	Ruminococcaceae	Clostridium	12	0.003	*	2	0.179	
Genus	Firmicutes	Clostridia	Ruminococcaceae	Faecalibacterium	13	0.002	***	3	0.112	
Genus	Firmicutes	Clostridia	Ruminococcaceae	Flavonifractor	15	0.001	***	2	0.200	
Genus	Firmicutes	Clostridia	Ruminococcaceae	Neglecta	3	0.201		1	0.389	
Genus	Firmicutes	Clostridia	Ruminococcaceae	Paludicola	20	0.000	***	2	0.200	
Genus	Firmicutes	Clostridia	Ruminococcaceae	Ruminococcaceae UCG-014	54	0.000	***	11	0.020	*
Genus	Firmicutes	Clostridia	Ruminococcaceae	Ruthenibacterium	6	0.054		3	0.166	
Genus	Firmicutes	Erysipelotrichia	Erysipelotrichaceae	Breznakia	36	0.000	***	2	0.200	
Genus	Firmicutes	Erysipelotrichia	Erysipelotrichaceae	Coprobacillus	14	0.001	***	0	0.917	
Genus	Fusobacteria	Fusobacteriia	Fusobacteriaceae	Fusobacterium	3	0.197		1	0.367	
Genus	Proteobacteria	Alphaproteobacteria	Rhodobacteraceae	Maritimibacter	10	0.008	**	2	0.200	
Genus	Proteobacteria	Deltaproteobacteria	Desulfovibrionaceae	Desulfovibrio	23	0.000	***	6	0.065	
Genus	Spirochaetes	Brachyspirae	Brachyspiraceae	Brachyspira	14	0.001	**	2	0.200	
Genus	Tenericutes	Mollicutes	Anaeroplasmataceae	Anaeroplasma	12	0.003	**	0	0.971	
Genus	Verrucomicrobia	Verrucomicrobiae	Akkermansiaceae	Akkermansia	50	0.000	***	12	0.013	*

Table S21: Differentially abundant ASV between regions in Mediterranean Siganidae

This table summarize the results of the Kruskal-Wallis tests comparing the CLR transformed abundances of bacterial ASVs between different regions.

					Siganus rivulatus			*Siganus luridus*		
**Rank**	**Phylum**	**Class**	**Family**	**Genus**	**Statistic**	**p-value corrected**		**Statistic**	**p-value corrected**	
Phylum	Bacteroidetes				10	0.010	**	17	0.011	*
Phylum	Deferribacteres				45	0.000	***	1	0.389	
Phylum	Epsilonbacteraeota				30	0.000	***	5	0.080	
Phylum	Firmicutes				69	0.000	***	14	0.011	*
Phylum	Fusobacteria				15	0.001	**	1	0.347	
Phylum	Proteobacteria				1	0.521		2	0.259	
Phylum	Spirochaetes				8	0.023	*	5	0.080	
Phylum	Tenericutes				28	0.000	***	6	0.071	
Phylum	Verrucomicrobia				40	0.000	***	4	0.104	
Family	Bacteroidetes	Bacteroidia	Marinifilaceae		4	0.135		1	0.410	
Family	Bacteroidetes	Bacteroidia	Marinilabiliaceae		39	0.000	***	0	0.543	
Family	Bacteroidetes	Bacteroidia	Rikenellaceae		3	0.212		13	0.011	**
Family	Bacteroidetes	Bacteroidia	Rs-E47 termite group		14	0.002	**	0	0.837	
Family	Bacteroidetes	Bacteroidia	Tannerellaceae		32	0.000	***	0	0.837	
Family	Deferribacteres	Deferribacteres	Deferribacteraceae		55	0.000	***	8	0.038	*
Family	Epsilonbacteraeota	Campylobacteria	Arcobacteraceae		40	0.000	***	7	0.038	*
Family	Firmicutes	Clostridia	Christensenellaceae		19	0.000	***	0	0.971	
Family	Firmicutes	Clostridia	Family XI		2	0.328		1	0.474	
Family	Firmicutes	Clostridia	Family XIII		16	0.001	***	0	0.699	
Family	Firmicutes	Clostridia	Lachnospiraceae		30	0.000	***	13	0.011	*
Family	Firmicutes	Clostridia	Ruminococcaceae		20	0.000	***	8	0.038	*
Family	Firmicutes	Erysipelotrichia	Erysipelotrichaceae		65	0.000	***	1	0.367	
Family	Fusobacteria	Fusobacteriia	Fusobacteriaceae		5	0.080		0	0.917	
Family	Proteobacteria	Alphaproteobacteria	Rhodobacteraceae		28	0.000	***	0	0.837	
Family	Proteobacteria	Deltaproteobacteria	Desulfovibrionaceae		34	0.000	***	11	0.024	*
Family	Spirochaetes	Brachyspirae	Brachyspiraceae		23	0.000	***	0	0.837	
Family	Tenericutes	Mollicutes	Anaeroplasmataceae		30	0.000	***	1	0.450	
Family	Verrucomicrobia	Verrucomicrobiae	Akkermansiaceae		41	0.000	***	8	0.038	*
Genus	Bacteroidetes	Bacteroidia	Marinifilaceae	Odoribacter	4	0.152		0	0.917	
Genus	Bacteroidetes	Bacteroidia	Marinilabiliaceae	Labilibacter	49	0.000	***	0	0.758	
Genus	Bacteroidetes	Bacteroidia	Rikenellaceae	Alistipes	37	0.000	***	5	0.077	
Genus	Bacteroidetes	Bacteroidia	Rikenellaceae	dgA-11 gut group	23	0.000	***	0	0.647	
Genus	Bacteroidetes	Bacteroidia	Rikenellaceae	Rikenella	2	0.394		2	0.243	
Genus	Bacteroidetes	Bacteroidia	Rikenellaceae	Rikenellaceae RC9 gut group	23	0.000	***	4	0.104	
Genus	Bacteroidetes	Bacteroidia	Tannerellaceae	Macellibacteroides	29	0.000	***	2	0.200	
Genus	Epsilonbacteraeota	Campylobacteria	Arcobacteraceae	Arcobacter	44	0.000	***	9	0.038	*
Genus	Firmicutes	Clostridia	Christensenellaceae	Christensenella	11	0.006	**	1	0.518	
Genus	Firmicutes	Clostridia	Family XI	Sedimentibacter	3	0.276		0	0.813	
Genus	Firmicutes	Clostridia	Lachnospiraceae	Bacteroides	14	0.001	***	0	0.868	
Genus	Firmicutes	Clostridia	Lachnospiraceae	Epulopiscium	55	0.000	***	2	0.200	
Genus	Firmicutes	Clostridia	Lachnospiraceae	Faecalicatena	12	0.003	**	2	0.229	
Genus	Firmicutes	Clostridia	Lachnospiraceae	Tyzzerella	8	0.022	*	0	0.647	
Genus	Firmicutes	Clostridia	Ruminococcaceae	Clostridium	12	0.003	*	2	0.179	
Genus	Firmicutes	Clostridia	Ruminococcaceae	Faecalibacterium	13	0.002	***	3	0.112	
Genus	Firmicutes	Clostridia	Ruminococcaceae	Flavonifractor	15	0.001	***	2	0.200	
Genus	Firmicutes	Clostridia	Ruminococcaceae	Neglecta	3	0.201		1	0.389	
Genus	Firmicutes	Clostridia	Ruminococcaceae	Paludicola	20	0.000	***	2	0.200	
Genus	Firmicutes	Clostridia	Ruminococcaceae	Ruminococcaceae UCG-014	54	0.000	***	11	0.020	*
Genus	Firmicutes	Clostridia	Ruminococcaceae	Ruthenibacterium	6	0.054		3	0.166	
Genus	Firmicutes	Erysipelotrichia	Erysipelotrichaceae	Breznakia	36	0.000	***	2	0.200	
Genus	Firmicutes	Erysipelotrichia	Erysipelotrichaceae	Coprobacillus	14	0.001	***	0	0.917	
Genus	Fusobacteria	Fusobacteriia	Fusobacteriaceae	Fusobacterium	3	0.197		1	0.367	
Genus	Proteobacteria	Alphaproteobacteria	Rhodobacteraceae	Maritimibacter	10	0.008	**	2	0.200	
Genus	Proteobacteria	Deltaproteobacteria	Desulfovibrionaceae	Desulfovibrio	23	0.000	***	6	0.065	
Genus	Spirochaetes	Brachyspirae	Brachyspiraceae	Brachyspira	14	0.001	**	2	0.200	
Genus	Tenericutes	Mollicutes	Anaeroplasmataceae	Anaeroplasma	12	0.003	**	0	0.971	
Genus	Verrucomicrobia	Verrucomicrobiae	Akkermansiaceae	Akkermansia	50	0.000	***	12	0.013	*

Table S22: Distance to centroid of environmental and Siganidae microbiomes in native and invaded range

Distance to centroids was estimated using the *betadisper()* function and using different dissimilarity indices.

			PERMDISP test			Distance to centroid		
**Diversity index**	**Ecosystem compartment**	**Rank**	**F-value**	**p-value**		**North Red Sea**	**Levantine Sea**	**Northern Crete**
Taxonomic q0	algae	ASV	9.5	0.003	**		0.49	0.56
Taxonomic q0	algae	Family	6.2	0.016	*		0.18	0.23
Taxonomic q0	algae	Phylum	5.4	0.024	*		0.09	0.13
Taxonomic q0	sediment	ASV	1.7	0.198		0.51		0.49
Taxonomic q0	sediment	Family	19.4	0.000	***	0.19		0.14
Taxonomic q0	sediment	Phylum	2.9	0.099		0.11		0.14
Taxonomic q0	turf	ASV	13.3	0.000	***	0.47	0.51	0.53
Taxonomic q0	turf	Family	8.1	0.001	***	0.15	0.24	0.20
Taxonomic q0	turf	Phylum	1.5	0.232		0.13	0.15	0.11
Taxonomic q0	water	ASV	2.0	0.153		0.32	0.39	0.33
Taxonomic q0	water	Family	3.4	0.043	*	0.24	0.20	0.20
Taxonomic q0	water	Phylum	0.3	0.766		0.20	0.18	0.18
Phylogenetic q0	*S. luridus*	ASV	2.4	0.134		0.23		0.17
Phylogenetic q0	*S. luridus*	Family	4.0	0.056		0.12		0.09
Phylogenetic q0	*S. luridus*	Phylum	0.7	0.411		0.07		0.06
Taxonomic q0	*S. luridus*	ASV	5.2	0.029	*	0.34		0.24
Taxonomic q0	*S. luridus*	Family	4.0	0.055		0.15		0.11
Taxonomic q0	*S. luridus*	Phylum	1.3	0.272		0.08		0.06
Phylogenetic q0	*S. rivulatus*	ASV	47.9	0.000	***	0.32	0.25	0.17
Phylogenetic q0	*S. rivulatus*	Family	33.0	0.000	***	0.16	0.08	0.05
Phylogenetic q0	*S. rivulatus*	Phylum	45.8	0.000	***	0.13	0.07	0.03
Taxonomic q0	*S. rivulatus*	ASV	62.1	0.000	***	0.42	0.34	0.23
Taxonomic q0	*S. rivulatus*	Family	24.1	0.000	***	0.19	0.09	0.07
Taxonomic q0	*S. rivulatus*	Phylum	38.3	0.000	***	0.15	0.08	0.04
Taxonomic q1	algae	ASV	9.1	0.004	**		0.42	0.52
Taxonomic q1	algae	Family	6.9	0.011	*		0.04	0.11
Taxonomic q1	algae	Phylum	0.1	0.713			0.02	0.03
Taxonomic q1	sediment	ASV	1.5	0.233		0.44		0.40
Taxonomic q1	sediment	Family	0.0	0.962		0.06		0.06
Taxonomic q1	sediment	Phylum	1.1	0.295		0.02		0.01
Taxonomic q1	turf	ASV	10.4	0.000	***	0.38	0.46	0.47
Taxonomic q1	turf	Family	9.0	0.001	***	0.04	0.15	0.06
Taxonomic q1	turf	Phylum	7.1	0.002	**	0.01	0.05	0.02
Taxonomic q1	water	ASV	0.4	0.659		0.22	0.22	0.24
Taxonomic q1	water	Family	1.2	0.310		0.07	0.06	0.08
Taxonomic q1	water	Phylum	4.3	0.022	*	0.01	0.01	0.03
Phylogenetic q1	*S. luridus*	ASV	5.5	0.026	*	0.14		0.09
Phylogenetic q1	*S. luridus*	Family	2.7	0.110		0.09		0.05
Phylogenetic q1	*S. luridus*	Phylum	2.0	0.170		0.07		0.05
Taxonomic q1	*S. luridus*	ASV	2.9	0.099		0.34		0.26
Taxonomic q1	*S. luridus*	Family	3.4	0.076		0.10		0.06
Taxonomic q1	*S. luridus*	Phylum	2.1	0.162		0.08		0.05
Phylogenetic q1	*S. rivulatus*	ASV	27.1	0.000	***	0.22	0.10	0.08
Phylogenetic q1	*S. rivulatus*	Family	25.9	0.000	***	0.18	0.07	0.06
Phylogenetic q1	*S. rivulatus*	Phylum	24.7	0.000	***	0.16	0.05	0.04
Taxonomic q1	*S. rivulatus*	ASV	35.8	0.000	***	0.44	0.32	0.25
Taxonomic q1	*S. rivulatus*	Family	23.9	0.000	***	0.20	0.09	0.07
Taxonomic q1	*S. rivulatus*	Phylum	24.7	0.000	***	0.16	0.05	0.04

Figure S8: Distance to centroid of Siganidae microbiomes in native and invaded range

Distance to centroids was estimated using the *betadisper()* function and using dissimilarity at the ASV level for four different indices: taxonomic and phylogenetic dissimilarity for both presence-absence and abundance-weighted data.

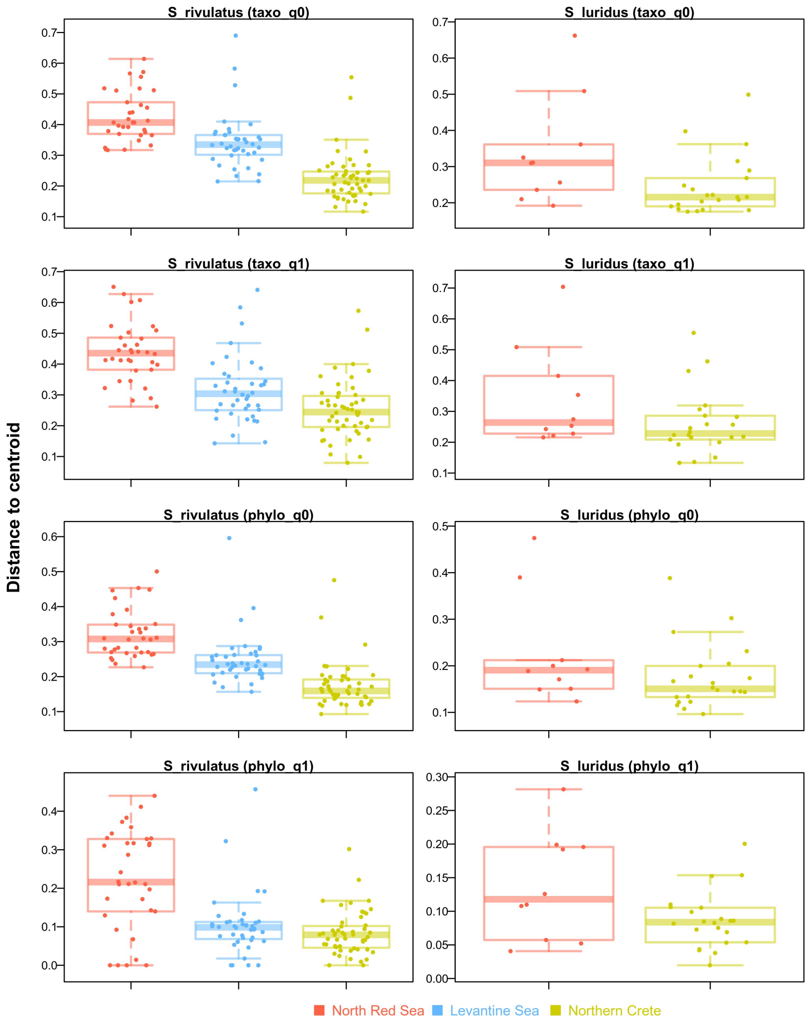

Figure S9: Distance to centroid of microbiomes from the environment in native and invaded range

Distance to centroids was estimated using the *betadisper()* function and using abundance-weighted dissimilarity at the ASV level.

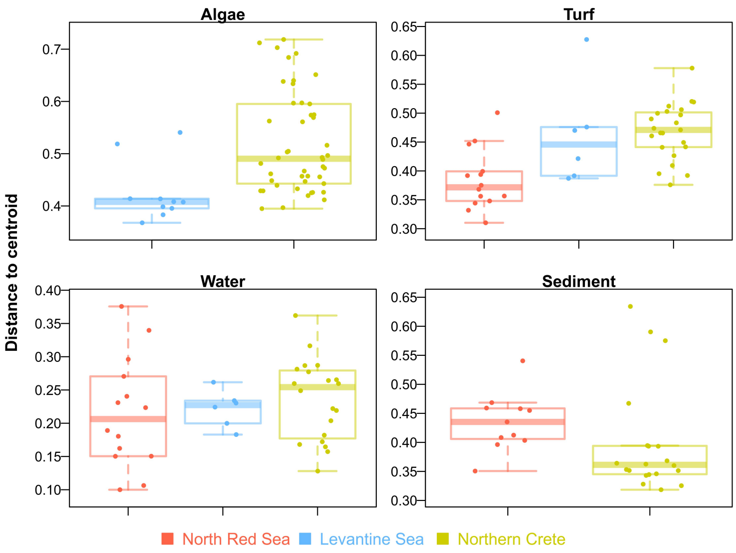

Table S23: Gut microbiome homogenization in Mediterranean Siganidae

This table contains the results of the non-parametric tests (Wilcoxon or Kruskal-Wallis) testing whether the dissimilarity intra-species changes between regions or whether dissimilarity inter-species changed between the native and invaded range. p values are FDR corrected and were all < 0.001.

				Average dissimilarity			% change in dissimilarity		
**Species**	**Diversity Facet**	**Rank**	**Dissimilarity index**	**North Red Sea**	**Levantine Sea**	**Northern Crete**	**Red Sea vs. Levantine Sea**	**Red Sea vs. Northern Crete**	**p-value**
Siganus rivulatus	Phylogenetic	ASV	q0	0.46	0.36	0.25	-22	-45	< 0.01
			q1	0.31	0.16	0.13	-50	-60	< 0.01
		Family	q0	0.22	0.11	0.07	-49	-66	< 0.01
			q1	0.25	0.11	0.09	-57	-65	< 0.01
		Phylum	q0	0.17	0.09	0.04	-48	-78	< 0.01
			q1	0.21	0.07	0.07	-66	-68	< 0.01
	Taxonomic	ASV	q0	0.60	0.50	0.33	-18	-46	< 0.01
			q1	0.61	0.45	0.37	-26	-40	< 0.01
		Family	q0	0.27	0.14	0.11	-49	-60	< 0.01
			q1	0.28	0.13	0.10	-54	-63	< 0.01
		Phylum	q0	0.21	0.11	0.05	-49	-75	< 0.01
			q1	0.22	0.07	0.07	-66	-68	< 0.01
Siganus luridus	Phylogenetic	ASV	q0	0.34		0.26		-24	< 0.01
			q1	0.20		0.13		-38	< 0.01
		Family	q0	0.18		0.13		-28	< 0.01
			q1	0.14		0.08		-41	< 0.01
		Phylum	q0	0.09		0.07		-14	< 0.01
			q1	0.11		0.07		-33	< 0.01
	Taxonomic	ASV	q0	0.34		0.26		-29	< 0.01
			q1	0.20		0.13		-26	< 0.01
		Family	q0	0.18		0.13		-31	< 0.01
			q1	0.14		0.08		-44	< 0.01
		Phylum	q0	0.09		0.07		-19	< 0.01
			q1	0.11		0.07		-33	< 0.01
Inter-species	Phylogenetic	ASV	q0	0.58		0.53		-9	< 0.01
			q1	0.33		0.20		-39	< 0.01
		Family	q0	0.24		0.17		-29	< 0.01
			q1	0.24		0.13		-46	< 0.01
		Phylum	q0	0.15		0.08		-42	< 0.01
			q1	0.17		0.11		-37	< 0.01
	Taxonomic	ASV	q0	0.81		0.69		-15	< 0.01
			q1	0.84		0.72		-15	< 0.01
		Family	q0	0.30		0.23		-24	< 0.01
			q1	0.28		0.15		-46	< 0.01
		Phylum	q0	0.18		0.10		-42	< 0.01
			q1	0.17		0.11		-38	< 0.01

Figure S10: Taxonomic dissimilarity of the gut microbiome of Mediterranean Siganidae across their native and invaded range

Taxonomic dissimilarity was assessed using partitioning of taxonomic entropy (using the Hill' numbers framework) computed on relative abundance of ASVs.

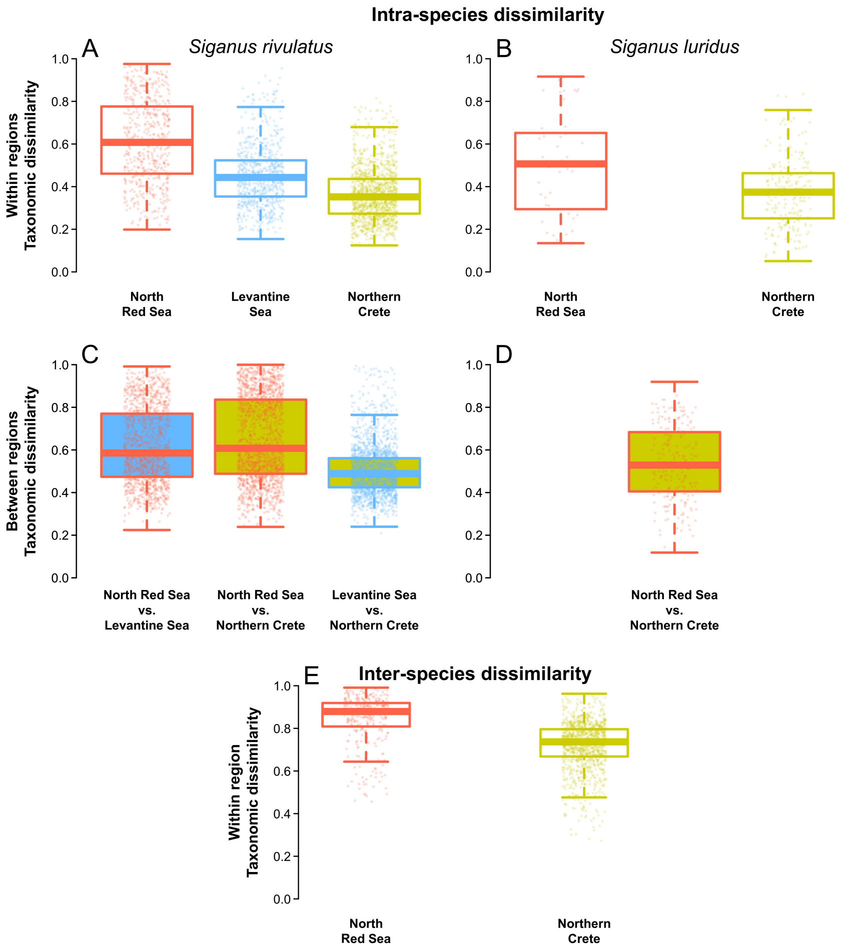

Figure S11: KEGG Orthologies with the highest contrast in abundance in the gut of *S. rivulatus* across the 3 regions

KEGG Orthologies ID are provided at top of each panel with letter in parentheses for the number of carbon atom in the corresponding short-chain fatty acids (SCFA). C1: formate; C2: acetate; C3: propionate; C4: butyrate; C5: valerate.

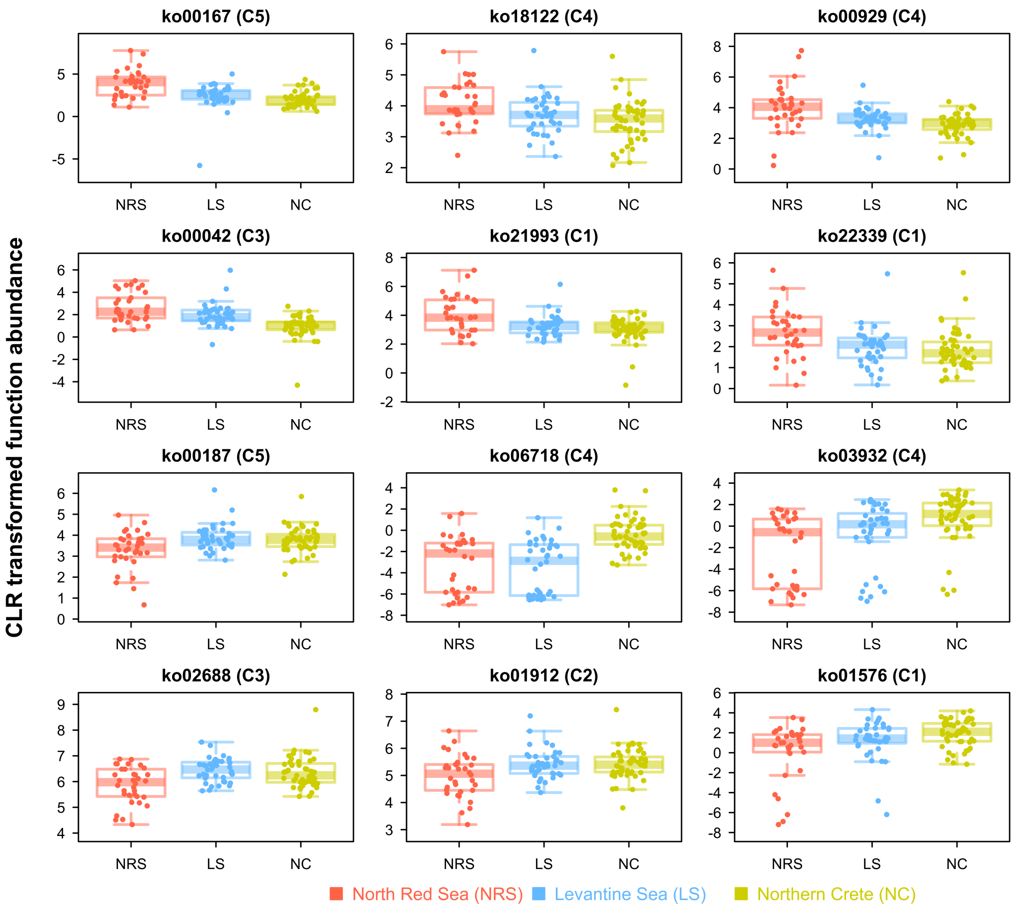

Table S24: Differentially abundant KEGG Orthologies between regions in Mediterranean Siganidae
This table summarize the results of the Kruskal-Wallis tests comparing the CLR transformed abundances of inferred KEGG Orthologies (KOs) between different regions.

		Siganus rivulatus				*Siganus luridus*			
**SCFA**	**# of C atoms**	**# of KO**	**Differentially abundant KO**		**Kruskal-Wallis test Mean statistic**	**# of KO**	**Differentially abundant KO**		**Kruskal-Wallis test Mean statistic**
			**#**	**%**			**#**	**%**	
Formate	1	25	14	56	10.0	25	2	8	2.6
Acetate	2	17	6	35	9.5	17	3	18	4.0
Propionate	3	13	10	77	20.3	13	4	31	4.8
Butyrate	4	17	10	59	16.3	17	8	47	4.9
Valerate	5	14	8	57	12.6	14	4	29	4.7

Table S25: Pariwise comparisons of the microbiome functional potential in Siganidae

This table summarizes the results of the PERMANOVA testing for pairwise differences between regions. These tests were performed using dissimilarity estimated on KOs relative abundance. The total number of test for each comparison is equal to 6 (*i.e.*KOs assocaited with SCFA with 1 to 5 C atoms and all KOs combined).

Species	Comparison	# of significant tests	average F-value	average p-value	average R2
S. luridus	Northern_Crete_vs_North_Red_Sea	6	18.4	0.001	0.37
S. rivulatus	Levantine_Sea_vs_North_Red_Sea	6	40.3	0.001	0.30
	Northern_Crete_vs_Levantine_Sea	4	5.2	0.067	0.05
	Northern_Crete_vs_North_Red_Sea	6	62.8	0.001	0.38

Table S26: Functional homogenization of the gut microbiome in Mediterranean Siganidae

This table contains the results of the non-parametric tests (Wilcoxon or Kruskal-Wallis) testing whether the dissimilarity intra-species changes between regions or whether dissimilarity inter-species changed between the native and invaded range. p-values are FDR corrected and were all < 0.001.

			Average dissimilarity			% change in dissimilarity		
Species	Type o SCFA	Dissimilarity index	North Red Sea	Levantine Sea	Northern Crete	Red Sea vs. Levantine Sea	Red Sea vs. Northern Crete	p-value
Siganus rivulatus	All SCFA	q0	0.14	0.09	0.05	-33	-66	<0.01
		q1	0.09	0.03	0.03	-71	-67	<0.01
	Formate (1C)	q0	0.13	0.11	0.05	-15	-66	<0.01
		q1	0.07	0.02	0.02	-71	-70	<0.01
	Acetate (2C)	q0	0.22	0.06	0.03	-73	-85	<0.01
		q1	0.09	0.02	0.03	-72	-64	<0.01
	Propionate (3C)	q0	0.10	0.10	0.03	4	-67	<0.01
		q1	0.07	0.02	0.02	-74	-75	<0.01
	Butyrate (4C)	q0	0.10	0.09	0.04	-16	-59	<0.01
		q1	0.10	0.04	0.05	-65	-48	<0.01
	Valerate (5C)	q0	0.21	0.12	0.10	-41	-50	<0.01
		q1	0.13	0.06	0.05	-58	-63	<0.01
Siganus luridus	All SCFA	q0	0.06		0.05		-22	<0.01
		q1	0.05		0.06		4	<0.01
	Formate (1C)	q0	0.06		0.07		17	<0.01
		q1	0.04		0.04		12	<0.01
	Acetate (2C)	q0	0.06		0.01		-78	<0.01
		q1	0.06		0.04		-32	<0.01
	Propionate (3C)	q0	0.09		0.04		-61	<0.01
		q1	0.03		0.04		21	<0.01
	Butyrate (4C)	q0	0.05		0.06		17	<0.01
		q1	0.06		0.09		41	<0.01
	Valerate (5C)	q0	0.08		0.07		-1	<0.01
		q1	0.08		0.08		-1	<0.01
Inter-species	All SCFA	q0	0.12		0.06		-52	<0.01
		q1	0.10		0.05		-50	<0.01
	Formate (1C)	q0	0.11		0.07		-38	<0.01
		q1	0.07		0.03		-49	<0.01
	Acetate (2C)	q0	0.15		0.02		-85	<0.01
		q1	0.09		0.04		-52	<0.01
	Propionate (3C)	q0	0.12		0.05		-54	<0.01
		q1	0.07		0.03		-52	<0.01
	Butyrate (4C)	q0	0.11		0.06		-48	<0.01
		q1	0.11		0.07		-33	<0.01
	Valerate (5C)	q0	0.16		0.10		-36	<0.01
		q1	0.16		0.07		-55	<0.01

Table S27: Functional distance to centroid of Siganidae microbiomes in native and invaded range

Distance to centroids was estimated using the *betadisper()* function and using dissimilarity at the KEGG Orthologies level for both presence-absence and abundance-weighted data.

			PERMDISP test			Distance to centroid		
Diversity index	Ecosystem compartment	SCFA	F-value	p-value		North Red Sea	Levantine Sea	Northern Crete
Functional q0	*S. luridus*	All SCFA	1.5	0.234		0.04		0.03
Functional q0	*S. luridus*	Formate	1.3	0.272		0.04		0.06
Functional q0	*S. luridus*	Acetate	9.8	0.004	**	0.04		0.01
Functional q0	*S. luridus*	Propionate	6.6	0.015	*	0.06		0.03
Functional q0	*S. luridus*	Butyrate	0.5	0.497		0.04		0.04
Functional q0	*S. luridus*	Valerate	0.0	0.964		0.05		0.05
Functional q0	*S. rivulatus*	All SCFA	25.9	0.000	***	0.09	0.07	0.03
Functional q0	*S. rivulatus*	Formate	16.0	0.000	***	0.09	0.09	0.04
Functional q0	*S. rivulatus*	Acetate	45.0	0.000	***	0.16	0.04	0.02
Functional q0	*S. rivulatus*	Propionate	17.3	0.000	***	0.07	0.07	0.03
Functional q0	*S. rivulatus*	Butyrate	25.5	0.000	***	0.08	0.06	0.03
Functional q0	*S. rivulatus*	Valerate	7.9	0.001	**	0.15	0.09	0.08
Functional q1	*S. luridus*	All SCFA	0.1	0.743		0.04		0.04
Functional q1	*S. luridus*	Formate	0.5	0.468		0.02		0.03
Functional q1	*S. luridus*	Acetate	0.8	0.384		0.04		0.03
Functional q1	*S. luridus*	Propionate	1.1	0.304		0.02		0.03
Functional q1	*S. luridus*	Butyrate	0.7	0.422		0.05		0.06
Functional q1	*S. luridus*	Valerate	0.0	0.843		0.05		0.05
Functional q1	*S. rivulatus*	All SCFA	27.2	0.000	***	0.06	0.02	0.02
Functional q1	*S. rivulatus*	Formate	25.1	0.000	***	0.05	0.01	0.01
Functional q1	*S. rivulatus*	Acetate	15.4	0.000	***	0.06	0.02	0.02
Functional q1	*S. rivulatus*	Propionate	20.0	0.000	***	0.04	0.01	0.01
Functional q1	*S. rivulatus*	Butyrate	10.6	0.000	***	0.07	0.02	0.03
Functional q1	*S. rivulatus*	Valerate	17.7	0.000	***	0.09	0.04	0.03

Figure S12: Functional dissimilarity of the gut microbiome of Mediterranean Siganidae across their native and invaded range

Functional dissimilarity was assessed using partitioning of functional entropy (using the Hill' numbers framework) computed on relative abundance of KEGG Orthologies associated with the metabolism of all short-chain fatty acids (SCFA; n = 86).

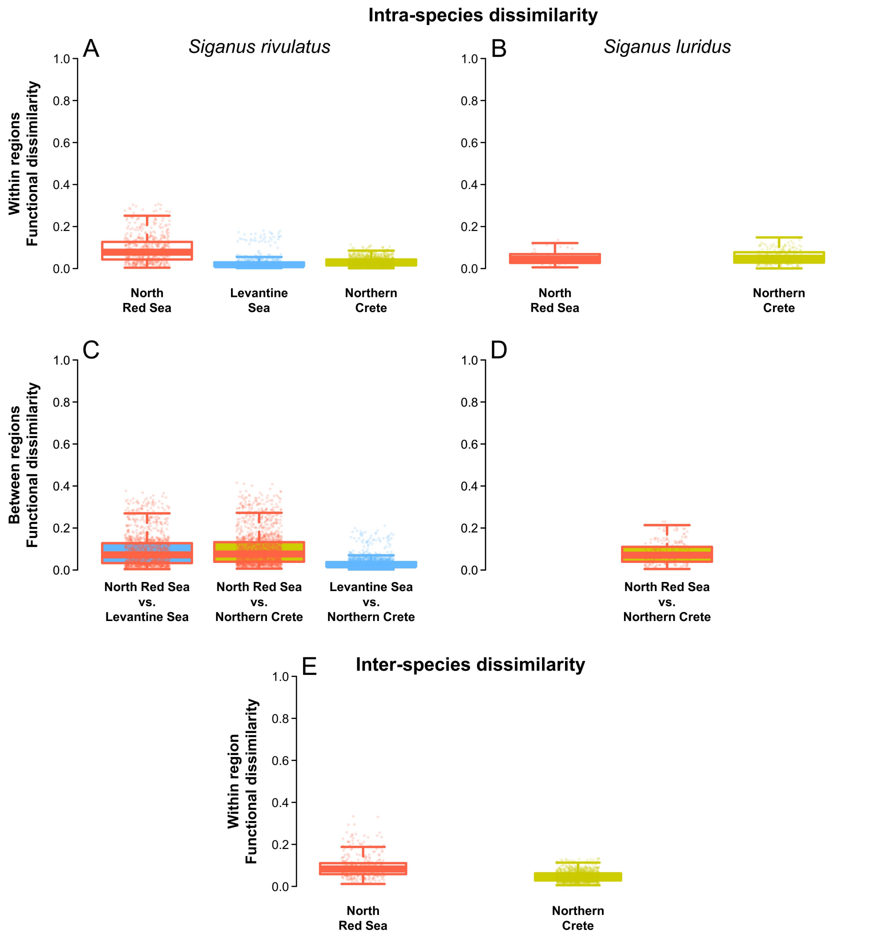

Table S28: List of KOs used in this study

KO	Enzyme_name	SCFA_C_number
ko01576	benzoylformate decarboxylase [ec:4.1.1.7]	C1
ko03385	nitrite reductase (cytochrome c-552) [ec:1.7.2.2]	C1
ko04014	protein nrfc	C1
ko04015	protein nrfd	C1
ko04016	cytochrome c-type biogenesis protein nrfe	C1
ko04069	pyruvate formate lyase activating enzyme [ec:1.97.1.4]	C1
ko04070	putative pyruvate formate lyase activating enzyme [ec:1.97.1.4]	C1
ko06212	formate transporter	C1
ko08177	mfs transporter, ofa family, oxalate/formate antiporter	C1
ko15828	formate hydrogenlyase subunit 3	C1
ko15829	formate hydrogenlyase subunit 4	C1
ko15830	formate hydrogenlyase subunit 5	C1
ko15831	formate hydrogenlyase subunit 6	C1
ko15832	formate hydrogenlyase subunit 7	C1
ko15834	formate hydrogenlyase maturation protein hych	C1
ko15836	formate hydrogenlyase transcriptional activator	C1
ko21636	ribonucleoside-triphosphate reductase (formate) [ec:1.1.98.6]	C1
ko21993	formate transporter	C1
ko22015	formate dehydrogenase (acceptor) [ec:1.17.99.7]	C1
ko22338	formate dehydrogenase (nad+, ferredoxin) subunit a [ec:1.17.1.11]	C1
ko22339	formate dehydrogenase (nad+, ferredoxin) subunit b [ec:1.17.1.11]	C1
ko22340	formate dehydrogenase (nad+, ferredoxin) subunit c [ec:1.17.1.11]	C1
ko22341	formate dehydrogenase (nad+, ferredoxin) subunit [ec:1.17.1.11]	C1
ko22515	formate dehydrogenase beta subunit [ec:1.17.1.9]	C1
ko22516	formate dehydrogenase (coenzyme f420) alpha subunit [ec:1.17.98.3 1.8.98.6]	C1
ko00483	4-hydroxyphenylacetate 3-monooxygenase [ec:1.14.14.9]	C2
ko00484	flavin reductase (nadh) [ec:1.5.1.36]	C2
ko01912	phenylacetate-coa ligase [ec:6.2.1.30]	C2
ko02610	ring-1,2-phenylacetyl-coa epoxidase subunit paab	C2
ko02613	ring-1,2-phenylacetyl-coa epoxidase subunit paae	C2
ko02614	acyl-coa thioesterase [ec:3.1.2.-]	C2
ko02616	phenylacetic acid degradation operon negative regulatory protein	C2
ko02618	oxepin-coa hydrolase / 3-oxo-5,6-dehydrosuberyl-coa semialdehyde dehydrogenase [ec:3.3.2.12 1.2.1.91]	C2
ko03150	2-iminoacetate synthase [ec:4.1.99.19]	C2
ko06193	protein phna	C2
ko13641	iclr family transcriptional regulator, acetate operon repressor	C2
ko14393	cation/acetate symporter	C2
ko18118	succinyl-coa:acetate coa-transferase [ec:2.8.3.18]	C2
ko19709	acetate coa-transferase [ec:2.8.3.8]	C2
ko21685	luxr family transcriptional regulator, regulator of acetate metabolism	C2
ko22027	indole-3-acetate monooxygenase [ec:1.14.13.235]	C2
ko22224	acetate---coa ligase (adp-forming) subunit beta [ec:6.2.1.13]	C2
ko00042	2-hydroxy-3-oxopropionate reductase [ec:1.1.1.60]	C3
ko00932	propionate kinase [ec:2.7.2.15]	C3
ko01505	1-aminocyclopropane-1-carboxylate deaminase [ec:3.5.99.7]	C3
ko01659	2-methylcitrate synthase [ec:2.3.3.5]	C3
ko01751	diaminopropionate ammonia-lyase [ec:4.3.1.15]	C3
ko02688	transcriptional regulator, propionate catabolism operon regulatory protein	C3
ko03416	methylmalonyl-coa carboxyltransferase 5s subunit [ec:2.1.3.1]	C3
ko03417	methylisocitrate lyase [ec:4.1.3.30]	C3
ko05712	3-(3-hydroxy-phenyl)propionate hydroxylase [ec:1.14.13.127]	C3
ko05820	mfs transporter, ppp family, 3-phenylpropionic acid transporter	C3
ko09022	2-iminobutanoate/2-iminopropanoate deaminase [ec:3.5.99.10]	C3
ko13923	phosphate propanoyltransferase [ec:2.3.1.222]	C3
ko19697	propionate kinase [ec:2.7.2.15]	C3
ko00004	(r,r)-butanediol dehydrogenase / meso-butanediol dehydrogenase / diacetyl reductase [ec:1.1.1.4 1.1.1.- 1.1.1.303]	C4
ko00020	3-hydroxyisobutyrate dehydrogenase [ec:1.1.1.31]	C4
ko00043	4-hydroxybutyrate dehydrogenase [ec:1.1.1.61]	C4
ko00606	3-methyl-2-oxobutanoate hydroxymethyltransferase [ec:2.1.2.11]	C4
ko00634	phosphate butyryltransferase [ec:2.3.1.19]	C4
ko00929	butyrate kinase [ec:2.7.2.7]	C4
ko03366	meso-butanediol dehydrogenase / (s,s)-butanediol dehydrogenase / diacetyl reductase [ec:1.1.1.- 1.1.1.76 1.1.1.304]	C4
ko03821	polyhydroxyalkanoate synthase [ec:2.3.1.-]	C4
ko03932	polyhydroxybutyrate depolymerase	C4
ko05973	poly(3-hydroxybutyrate) depolymerase [ec:3.1.1.75]	C4
ko06718	l-2,4-diaminobutyric acid acetyltransferase [ec:2.3.1.178]	C4
ko13745	l-2,4-diaminobutyrate decarboxylase [ec:4.1.1.86]	C4
ko15784	n2-acetyl-l-2,4-diaminobutanoate deacetylase [ec:3.5.1.125]	C4
ko15785	l-2,4-diaminobutyrate transaminase [ec:2.6.1.76]	C4
ko16871	4-aminobutyrate---pyruvate transaminase [ec:2.6.1.96]	C4
ko18120	4-hydroxybutyrate dehydrogenase [ec:1.1.1.61]	C4
ko18122	4-hydroxybutyrate coa-transferase [ec:2.8.3.-]	C4
ko00166	2-oxoisovalerate dehydrogenase e1 component alpha subunit [ec:1.2.4.4]	C5
ko00167	2-oxoisovalerate dehydrogenase e1 component beta subunit [ec:1.2.4.4]	C5
ko00186	2-oxoisovalerate ferredoxin oxidoreductase, alpha subunit [ec:1.2.7.7]	C5
ko00187	2-oxoisovalerate ferredoxin oxidoreductase, beta subunit [ec:1.2.7.7]	C5
ko00188	2-oxoisovalerate ferredoxin oxidoreductase, delta subunit [ec:1.2.7.7]	C5
ko09699	2-oxoisovalerate dehydrogenase e2 component (dihydrolipoyl transacylase) [ec:2.3.1.168]	C5
ko11381	2-oxoisovalerate dehydrogenase e1 component [ec:1.2.4.4]	C5
ko13877	2,5-dioxopentanoate dehydrogenase [ec:1.2.1.26]	C5
ko18365	4-hydroxy-2-oxovalerate/4-hydroxy-2-oxohexanoate aldolase [ec:4.1.3.39 4.1.3.43]	C5
ko19588	2,5-dioxopentanoate dehydrogenase [ec:1.2.1.26]	C5
ko20882	(r)-2-hydroxy-4-methylpentanoate coa-transferase [ec:2.8.3.24]	C5
ko21399	2-amino-4-ketopentanoate thiolase alpha subunit [ec:2.3.1.263]	C5
ko21400	2-amino-4-ketopentanoate thiolase beta subunit [ec:2.3.1.263]	C5
ko21672	2,4-diaminopentanoate dehydrogenase [ec:1.4.1.12 1.4.1.26]	C5

Table S29: Biological information on the fishes used in this study

sample_id	sample_type	taxonomy	region	season	body_mass	standard_length	size_class	sex_state	gut_fullness
I_F039	Fish	Siganus_rivulatus	Levantine_Sea	Spring	67	147	[100,200]	Female	full
I_F040	Fish	Siganus_rivulatus	Levantine_Sea	Spring	203	192	[100,200]	Male	only_intestine
I_F041	Fish	Siganus_rivulatus	Levantine_Sea	Spring	67	140	[100,200]	Male	only_intestine
I_F043	Fish	Siganus_rivulatus	Levantine_Sea	Spring	98	163	[100,200]	Female	two_third_full
I_F046	Fish	Siganus_rivulatus	Levantine_Sea	Spring	88	165	[100,200]	Male	full
I_F047	Fish	Siganus_rivulatus	Levantine_Sea	Spring	71	157	[100,200]	Male	full
I_F049	Fish	Siganus_rivulatus	Levantine_Sea	Spring	88	155	[100,200]	Female	full
I_F053	Fish	Siganus_rivulatus	Levantine_Sea	Spring	70	155	[100,200]	Male	full
I_F054	Fish	Siganus_rivulatus	Levantine_Sea	Spring	78	155	[100,200]	Female	full
I_F055	Fish	Siganus_rivulatus	Levantine_Sea	Spring	36	118	[100,200]	Male	full
I_F056	Fish	Siganus_rivulatus	Levantine_Sea	Spring	73	156	[100,200]	Male	full
I_F057	Fish	Siganus_rivulatus	Levantine_Sea	Spring	78	153	[100,200]	Male	full
I_F058	Fish	Siganus_rivulatus	Levantine_Sea	Spring	123	178	[100,200]	Female	full
I_F059	Fish	Siganus_rivulatus	Levantine_Sea	Spring	58	121	[100,200]	Female	full
I_F060	Fish	Siganus_rivulatus	Levantine_Sea	Spring	130	177	[100,200]	Female	full
I_F061	Fish	Siganus_rivulatus	Levantine_Sea	Spring	116	173	[100,200]	Male	full
I_F062	Fish	Siganus_rivulatus	Levantine_Sea	Spring	50	135	[100,200]	Male	full
I_F063	Fish	Siganus_rivulatus	Levantine_Sea	Spring	56	137	[100,200]	Male	full
I_F064	Fish	Siganus_rivulatus	Levantine_Sea	Spring	48	130	[100,200]	Male	full
I_F065	Fish	Siganus_rivulatus	Levantine_Sea	Spring	54	137	[100,200]	Male	full
I_F066	Fish	Siganus_rivulatus	Levantine_Sea	Spring	107	172	[100,200]	Female	full
I_F067	Fish	Siganus_rivulatus	Levantine_Sea	Spring	51	143	[100,200]	Male	full
I_F068	Fish	Siganus_rivulatus	Levantine_Sea	Spring	67	147	[100,200]	Male	full
I_F375	Fish	Siganus_rivulatus	Levantine_Sea	Autumn	31	105	[100,200]	NA	full
I_F376	Fish	Siganus_rivulatus	Levantine_Sea	Autumn	27	107	[100,200]	NA	full
I_F377	Fish	Siganus_rivulatus	Levantine_Sea	Autumn	62	142	[100,200]	NA	full
I_F378	Fish	Siganus_rivulatus	Levantine_Sea	Autumn	75	154	[100,200]	NA	full
I_F379	Fish	Siganus_rivulatus	Levantine_Sea	Autumn	47	134	[100,200]	NA	half_full
I_F381	Fish	Siganus_rivulatus	Levantine_Sea	Autumn	40	124	[100,200]	NA	half_full
I_F382	Fish	Siganus_rivulatus	Levantine_Sea	Autumn	20	112	[100,200]	NA	two_third_full
I_F384	Fish	Siganus_rivulatus	Levantine_Sea	Autumn	22	115	[100,200]	NA	half_full
I_F389	Fish	Siganus_rivulatus	Levantine_Sea	Autumn	56	141	[100,200]	NA	half_full
I_F395	Fish	Siganus_rivulatus	Levantine_Sea	Autumn	31	111	[100,200]	NA	two_third_full
I_F399	Fish	Siganus_rivulatus	Levantine_Sea	Autumn	89	161	[100,200]	NA	full
I_F401	Fish	Siganus_rivulatus	Levantine_Sea	Autumn	60	144	[100,200]	NA	full
I_F403	Fish	Siganus_rivulatus	Levantine_Sea	Autumn	43	121	[100,200]	NA	three_quarter_full
I_F406	Fish	Siganus_rivulatus	Levantine_Sea	Autumn	45	128	[100,200]	NA	two_third_full
I_F407	Fish	Siganus_rivulatus	Levantine_Sea	Autumn	34	118	[100,200]	NA	half_full
I_F408	Fish	Siganus_rivulatus	Levantine_Sea	Autumn	52	129	[100,200]	NA	two_third_full
I_F409	Fish	Siganus_rivulatus	Levantine_Sea	Autumn	44	129	[100,200]	NA	two_third_full
R_F001	Fish	Siganus_rivulatus	North_Red_Sea	Spring	203	190	[100,200]	M	half_full
R_F002	Fish	Siganus_rivulatus	North_Red_Sea	Spring	234	192	[100,200]	Female	half_full
R_F004	Fish	Siganus_luridus	North_Red_Sea	Spring	440	235	> 200	Female	full
R_F005	Fish	Siganus_luridus	North_Red_Sea	Spring	295	206	> 200	Female	full
R_F006	Fish	Siganus_rivulatus	North_Red_Sea	Spring	281	220	> 200	Female	full
R_F007	Fish	Siganus_rivulatus	North_Red_Sea	Spring	142	200	[100,200]	Female	full
R_F013	Fish	Siganus_rivulatus	North_Red_Sea	Spring	196	205	> 200	Female	full
R_F014	Fish	Siganus_rivulatus	North_Red_Sea	Spring	168	193	[100,200]	Female	full
R_F015	Fish	Siganus_rivulatus	North_Red_Sea	Spring	326	245	> 200	Female	full
R_F016	Fish	Siganus_rivulatus	North_Red_Sea	Spring	200	200	[100,200]	Female	full
R_F017	Fish	Siganus_rivulatus	North_Red_Sea	Spring	82	155	[100,200]	Immature	full
R_F018	Fish	Siganus_luridus	North_Red_Sea	Spring	148	182	[100,200]	Female	full
R_F019	Fish	Siganus_luridus	North_Red_Sea	Spring	142	185	[100,200]	Male	full
R_F020	Fish	Siganus_luridus	North_Red_Sea	Spring	163	199	[100,200]	Female	full
R_F021	Fish	Siganus_rivulatus	North_Red_Sea	Spring	272	240	> 200	Female	NA
R_F024	Fish	Siganus_luridus	North_Red_Sea	Spring	125	178	[100,200]	Female	NA
R_F025	Fish	Siganus_luridus	North_Red_Sea	Spring	173	200	[100,200]	Female	NA
R_F029	Fish	Siganus_rivulatus	North_Red_Sea	Spring	339	245	> 200	Female	full
R_F030	Fish	Siganus_rivulatus	North_Red_Sea	Spring	51	130	[100,200]	Immature	full
R_F032	Fish	Siganus_rivulatus	North_Red_Sea	Spring	41	123	[100,200]	Immature	only_intestine
R_F033	Fish	Siganus_rivulatus	North_Red_Sea	Spring	60	135	[100,200]	Immature	only_intestine
R_F034	Fish	Siganus_rivulatus	North_Red_Sea	Spring	33	120	[100,200]	Immature	only_intestine
R_F035	Fish	Siganus_rivulatus	North_Red_Sea	Spring	36	120	[100,200]	Immature	only_intestine
R_F036	Fish	Siganus_rivulatus	North_Red_Sea	Spring	22	102	[100,200]	Immature	only_intestine
R_F334	Fish	Siganus_rivulatus	North_Red_Sea	Autumn	154	208	> 200	NA	two_third_full
R_F335	Fish	Siganus_luridus	North_Red_Sea	Autumn	188	197	[100,200]	Female	three_quarter_full
R_F336	Fish	Siganus_rivulatus	North_Red_Sea	Autumn	190	212	> 200	NA	full
R_F337	Fish	Siganus_luridus	North_Red_Sea	Autumn	210	203	> 200	Female	two_third_full
R_F338	Fish	Siganus_luridus	North_Red_Sea	Autumn	61	155	[100,200]	Female	half_full
R_F339	Fish	Siganus_rivulatus	North_Red_Sea	Autumn	115	218	> 200	NA	two_third_full
R_F340	Fish	Siganus_rivulatus	North_Red_Sea	Autumn	188	216	> 200	NA	half_full
R_F341	Fish	Siganus_rivulatus	North_Red_Sea	Autumn	76	155	[100,200]	NA	half_full
R_F342	Fish	Siganus_rivulatus	North_Red_Sea	Autumn	203	235	> 200	NA	half_full
R_F343	Fish	Siganus_rivulatus	North_Red_Sea	Autumn	176	205	> 200	NA	half_full
R_F344	Fish	Siganus_rivulatus	North_Red_Sea	Autumn	146	179	[100,200]	NA	half_full
R_F345	Fish	Siganus_rivulatus	North_Red_Sea	Autumn	75	162	[100,200]	NA	half_full
R_F353	Fish	Siganus_rivulatus	North_Red_Sea	Autumn	127	167	[100,200]	NA	half_full
R_F354	Fish	Siganus_rivulatus	North_Red_Sea	Autumn	45	111	[100,200]	NA	half_full
R_F355	Fish	Siganus_rivulatus	North_Red_Sea	Autumn	82	140	[100,200]	NA	two_third_full

sample_id	sample_type	taxonomy	region	season	body_mass	standard_length	size_class	sex_state	gut_fullness
R_F356	Fish	Siganus_rivulatus	North_Red_Sea	Autumn	159	179	[100,200]	NA	two_third_full
R_F357	Fish	Siganus_rivulatus	North_Red_Sea	Autumn	55	135	[100,200]	NA	two_third_full
R_F360	Fish	Siganus_rivulatus	North_Red_Sea	Autumn	55	136	[100,200]	NA	three_quarter_full
R_F361	Fish	Siganus_rivulatus	North_Red_Sea	Autumn	50	132	[100,200]	NA	half_full
G_F612	Fish	Siganus_rivulatus	Northern_Crete	Spring	91	154	[100,200]	Female	full
G_F615	Fish	Siganus_rivulatus	Northern_Crete	Spring	71	153	[100,200]	Female	full
G_F617	Fish	Siganus_rivulatus	Northern_Crete	Spring	122	187	[100,200]	Female	full
G_F618	Fish	Siganus_rivulatus	Northern_Crete	Spring	84	145	[100,200]	Immature	full
G_F620	Fish	Siganus_rivulatus	Northern_Crete	Spring	76	146	[100,200]	Male	full
G_F622	Fish	Siganus_rivulatus	Northern_Crete	Spring	85	149	[100,200]	Female	full
G_F623	Fish	Siganus_rivulatus	Northern_Crete	Spring	124	183	[100,200]	Male	full
G_F625	Fish	Siganus_rivulatus	Northern_Crete	Spring	123	183	[100,200]	Male	full
G_F631	Fish	Siganus_luridus	Northern_Crete	Spring	61	134	[100,200]	Female	NA
G_F632	Fish	Siganus_rivulatus	Northern_Crete	Spring	118	173	[100,200]	Male	first_and_filled_last_quarter
G_F633	Fish	Siganus_luridus	Northern_Crete	Spring	77	132	[100,200]	NA	full
G_F634	Fish	Siganus_luridus	Northern_Crete	Spring	61	122	[100,200]	Male	full
G_F635	Fish	Siganus_luridus	Northern_Crete	Spring	77	143	[100,200]	NA	full
G_F636	Fish	Siganus_luridus	Northern_Crete	Spring	66	125	[100,200]	Female	full
G_F642	Fish	Siganus_rivulatus	Northern_Crete	Spring	183	220	> 200	Female	full
G_F645	Fish	Siganus_rivulatus	Northern_Crete	Spring	52	128	[100,200]	Male	full
G_F647	Fish	Siganus_luridus	Northern_Crete	Spring	74	129	[100,200]	Male	full
G_F665	Fish	Siganus_rivulatus	Northern_Crete	Spring	162	184	[100,200]	Female	full
G_F666	Fish	Siganus_rivulatus	Northern_Crete	Spring	109	174	[100,200]	Female	full
G_F667	Fish	Siganus_rivulatus	Northern_Crete	Spring	112	172	[100,200]	Female	NA
G_F669	Fish	Siganus_rivulatus	Northern_Crete	Spring	56	130	[100,200]	Male	NA
G_F670	Fish	Siganus_rivulatus	Northern_Crete	Spring	44	122	[100,200]	Immature	NA
G_F674	Fish	Siganus_rivulatus	Northern_Crete	Spring	35	115	[100,200]	Immature	NA
G_F675	Fish	Siganus_rivulatus	Northern_Crete	Spring	82	153	[100,200]	Female	NA
G_F676	Fish	Siganus_rivulatus	Northern_Crete	Spring	99	164	[100,200]	Male	NA
G_F686	Fish	Siganus_rivulatus	Northern_Crete	Spring	40	124	[100,200]	NA	full
G_F687	Fish	Siganus_rivulatus	Northern_Crete	Spring	94	153	[100,200]	Female	full
G_F690	Fish	Siganus_rivulatus	Northern_Crete	Spring	62	135	[100,200]	Male	full
G_F709	Fish	Siganus_rivulatus	Northern_Crete	Spring	145	185	[100,200]	Female	full
G_F723	Fish	Siganus_rivulatus	Northern_Crete	Spring	95	176	[100,200]	Female	filled_last_quarter
G_F724	Fish	Siganus_rivulatus	Northern_Crete	Spring	77	153	[100,200]	Male	filled_last_quarter
G_F725	Fish	Siganus_rivulatus	Northern_Crete	Spring	91	174	[100,200]	Male	filled_last_quarter
G_F726	Fish	Siganus_luridus	Northern_Crete	Spring	64	136	[100,200]	Female	filled_last_quarter
G_F727	Fish	Siganus_luridus	Northern_Crete	Spring	90	145	[100,200]	Male	filled_last_quarter
G_F728	Fish	Siganus_luridus	Northern_Crete	Spring	96	145	[100,200]	Female	almost_full
G_F729	Fish	Siganus_luridus	Northern_Crete	Spring	89	142	[100,200]	Male	first_and_filled_last_quarter
G_F730	Fish	Siganus_luridus	Northern_Crete	Spring	74	138	[100,200]	Female	second_and_filled_last_quarter
G_F731	Fish	Siganus_rivulatus	Northern_Crete	Spring	104	176	[100,200]	Male	filled_last_quarter
G_F1004	Fish	Siganus_luridus	Northern_Crete	Autumn	20	103	[100,200]	Immature	NA
G_F1006	Fish	Siganus_luridus	Northern_Crete	Autumn	19	104	[100,200]	Immature	full
G_F868	Fish	Siganus_rivulatus	Northern_Crete	Autumn	140	175	[100,200]	Immature	full
G_F869	Fish	Siganus_rivulatus	Northern_Crete	Autumn	109	155	[100,200]	Immature	full
G_F871	Fish	Siganus_rivulatus	Northern_Crete	Autumn	85	147	[100,200]	Immature	full
G_F873	Fish	Siganus_rivulatus	Northern_Crete	Autumn	102	156	[100,200]	Immature	full
G_F874	Fish	Siganus_rivulatus	Northern_Crete	Autumn	114	157	[100,200]	Immature	full
G_F875	Fish	Siganus_rivulatus	Northern_Crete	Autumn	42	117	[100,200]	Immature	full
G_F876	Fish	Siganus_rivulatus	Northern_Crete	Autumn	44	124	[100,200]	Immature	full
G_F883	Fish	Siganus_rivulatus	Northern_Crete	Autumn	NA	135	[100,200]	Immature	full
G_F885	Fish	Siganus_rivulatus	Northern_Crete	Autumn	51	126	[100,200]	Immature	full
G_F886	Fish	Siganus_rivulatus	Northern_Crete	Autumn	56	135	[100,200]	Immature	full
G_F887	Fish	Siganus_rivulatus	Northern_Crete	Autumn	82	114	[100,200]	Immature	full
G_F899	Fish	Siganus_rivulatus	Northern_Crete	Autumn	69	127	[100,200]	Immature	full
G_F901	Fish	Siganus_rivulatus	Northern_Crete	Autumn	85	140	[100,200]	Immature	full
G_F913	Fish	Siganus_rivulatus	Northern_Crete	Autumn	NA	160	[100,200]	Immature	full
G_F915	Fish	Siganus_rivulatus	Northern_Crete	Autumn	155	210	> 200	NA	full
G_F931	Fish	Siganus_luridus	Northern_Crete	Autumn	78	132	[100,200]	Male	fat_gut_full
G_F933	Fish	Siganus_rivulatus	Northern_Crete	Autumn	79	150	[100,200]	Immature	fat_gut_full
G_F934	Fish	Siganus_rivulatus	Northern_Crete	Autumn	46	127	[100,200]	Immature	full
G_F941	Fish	Siganus_rivulatus	Northern_Crete	Autumn	113	160	[100,200]	Immature	fat_gut_full
G_F942	Fish	Siganus_rivulatus	Northern_Crete	Autumn	99	155	[100,200]	NA	fat_gut_full
G_F947	Fish	Siganus_luridus	Northern_Crete	Autumn	97	182	[100,200]	NA	full
G_F953	Fish	Siganus_luridus	Northern_Crete	Autumn	93	170	[100,200]	Immature	full
G_F954	Fish	Siganus_luridus	Northern_Crete	Autumn	81	165	[100,200]	Immature	full
G_F958	Fish	Siganus_luridus	Northern_Crete	Autumn	133	186	[100,200]	Immature	full
G_F959	Fish	Siganus_luridus	Northern_Crete	Autumn	108	180	[100,200]	NA	full
G_F960	Fish	Siganus_luridus	Northern_Crete	Autumn	108	172	[100,200]	Immature	full
G_F965	Fish	Siganus_rivulatus	Northern_Crete	Autumn	137	187	[100,200]	Immature	full
G_F968	Fish	Siganus_luridus	Northern_Crete	Autumn	83	170	[100,200]	Immature	full
G_F970	Fish	Siganus_rivulatus	Northern_Crete	Autumn	43	143	[100,200]	Immature	full
G_F973	Fish	Siganus_rivulatus	Northern_Crete	Autumn	126	186	[100,200]	Immature	full
G_F974	Fish	Siganus_rivulatus	Northern_Crete	Autumn	20	108	[100,200]	Immature	full
G_F976	Fish	Siganus_rivulatus	Northern_Crete	Autumn	100	181	[100,200]	Immature	full
G_F979	Fish	Siganus_rivulatus	Northern_Crete	Autumn	72	170	[100,200]	Immature	full
G_F999	Fish	Siganus_luridus	Northern_Crete	Autumn	83	138	[100,200]	Immature	full
